# Synergistic Construction of In Situ Self‐Polymerized Interface and Localized pH Buffer Zone for High‐Performance Aqueous Zinc–Iodine Batteries

**DOI:** 10.1002/anie.202511490

**Published:** 2025-08-27

**Authors:** Jiapei Li, Zhiying Fang, Hanjian Chen, Kunlun Liu, Yicai Pan, Xiaoge Li, Dewu Lin, Nanyang Wang, Can Guo, Cuiping Han, Yagang Yao, Pan Xue, Guo Hong

**Affiliations:** ^1^ Department of Materials Science and Engineering & Center of Super‐Diamond and Advanced Films (COSDAF) City University of Hong Kong Hong Kong 999077 P.R. China; ^2^ School of Chemistry and Chemical Engineering Yangzhou University Yangzhou 225009 P.R. China; ^3^ State Key Laboratory of Advanced Waterproof Materials School of Advanced Materials Shenzhen Graduate School Peking University Shenzhen 518055 P.R. China; ^4^ NTI‐NTU Corporate Laboratory Nanyang Technological University Singapore 637662 Singapore; ^5^ Department of Applied Biology and Chemical Technology Faculty of Science The Hong Kong Polytechnic University Hong Kong 999077 P.R. China; ^6^ Faculty of Materials Science and Energy Engineering Shenzhen University of Advanced Technology Shenzhen 518107 P.R. China; ^7^ National Laboratory of Solid State Microstructures College of Engineering and Applied Sciences Jiangsu Key Laboratory of Artificial Functional Materials Collaborative Innovation Center of Advanced Microstructures Nanjing University Nanjing 210023 P.R. China; ^8^ The Shenzhen Research Institute City University of Hong Kong Shenzhen 518057 P.R. China

**Keywords:** Inhibition of polyiodide shuttle effect, Organic pH buffer, Stable in situ SEI, Suppression of Zn dendrite growth

## Abstract

Aqueous zinc–iodine (Zn–I_2_) batteries are promising for large‐scale energy storage. However, their practical use is hindered by challenges such as Zn dendrite growth, hydrogen evolution reaction (HER), corrosion, and polyiodide shuttle effect. In this study, valerolactam (VL) is employed as an organic pH buffer to address these issues. Theoretical and experimental results demonstrate that VL can regulate the electrolyte local pH while in situ polymerizing on the electrode surface to form a mechanically stable solid electrolyte interphase (SEI) protection layer, effectively suppressing HER, corrosion, and dendrite growth. Furthermore, the introduction of VL significantly regulates the solvation structure of Zn^2+^, and disrupts the inherent hydrogen bonding network, which enhances the electrochemical performance. As a result, a symmetric cell with VL‐based electrolyte achieves impressive longevity under ultra‐high current density (4000 cycles at 40 mA cm^−2^ and 1 mAh cm^−2^), 4.3 times higher than the counterpart in the conventional ZnSO_4_ electrolytes. Moreover, VL effectively suppresses polyiodide shuttle effect and improves electrochemical stability. Consequently, Zn–I_2_ full battery exhibits exceptional cycling stability, sustaining 26 500 cycles with a high‐capacity retention of 86.4%. Therefore, organic pH buffering engineering has been proved to be a promising strategy for achieving dendrite‐free, shuttle‐free Zn–I_2_ batteries.

## Graphical abstract

A multifunctional electrolyte pH buffer, valerolactam, was developed to stabilize the zinc anode, regulate interfacial pH, self‐polymerize into a robust SEI layer, and suppress polyiodide shuttling, thereby significantly enhancing the performance of aqueous Zn–I_2_ batteries.

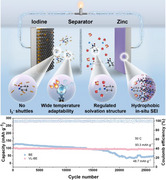

## Introduction

Aqueous zinc–iodine (Zn–I_2_) batteries have emerged as ideal candidates for large‐scale energy storage and transmission due to their high safety, nontoxicity, and low cost.^[^ ^1^, ^2^, ^3^, ^4^, ^5^ ^]^ However, unavoidable side reactions, such as hydrogen evolution reaction (HER), corrosion, and uncontrolled Zn deposition at the electrolyte/anode interface, severely hinder their further commercialization.^[^ ^6^, ^7^, ^8^ ^]^ These side reactions originate from the inherent thermodynamic instability of aqueous Zn–I_2_ batteries, leading to a continuous increase in electrolyte pH, which subsequently induces the uncontrolled formation of by‐products and dendrites.^[^ ^9^, ^10^, ^11^, ^12^ ^]^ The stress and strain caused by these dendrites and by‐products can disrupt the solid electrolyte interphase (SEI), further exacerbating various uncontrolled side reactions within the battery.^[^ ^13^, ^14^, ^15^, ^16^, ^17^, ^18^, ^19^ ^]^ Regulating the local pH at the electrode–electrolyte interface helps stabilize fluctuations in the overall electrolyte pH. Therefore, developing simple and universal strategies to regulate the local pH to suppress the continuous formation of dendrites and by‐products is of great significance for achieving a high‐performance, dendrite‐free, and side‐reaction‐free Zn anode.

To address these challenges, researchers have proposed the in situ construction of multifunctional SEI protective layers on the electrode surface to regulate local pH, thereby suppressing side reactions while promoting uniform Zn deposition.^[^ ^20^ ^]^ These strategies mainly include the artificial modification of anode/electrolyte interface^[^ ^21^, ^22^, ^23^, ^24^ ^]^ and the development of electrolyte additives.^[^ ^25^, ^26^, ^27^, ^28^ ^]^ Among them, modifying the electrolyte to regulate local pH has attracted extensive attention due to its simplicity and broad applicability.^[^ ^9^, ^29^ ^]^ Common electrolyte pH buffers can be categorized into weak acid ammonium salts and nitrogen‐containing heterocyclic organic compounds.^[^ ^12^, ^30^ ^]^ For example, He et al. proposed ammonium dihydrogen phosphate as a multifunctional additive, which regulated local pH through the interconversion of weak acid ions, promoted highly reversible Zn plating/stripping and suppressed side reactions during cycling.^[^ ^12^ ^]^ Guo et al. employed nitrogen‐containing heterocyclic compounds as organic pH buffers, such as pyridine and imidazole, which regulated interfacial pH through the adsorption and release of H^+^ at pyridine N sites, thereby inhibiting HER and anode corrosion.^[^ ^30^ ^]^ However, due to their high structural stability, these additives are difficult to be decomposed on the Zn anode surface to form an in situ SEI layer, leading to poor SEI structural stability.^[^ ^31^ ^]^ Therefore, optimizing electrolyte composition to dynamically regulate the local pH and promote the in situ polymerization of a stable SEI layer is crucial to improving battery stability and cycling performance.

In this study, we have employed valerolactam (VL), a cyclic organic molecule containing an amide group, as a multifunctional electrolyte co‐solvent and pH buffer to tackle the aforesaid problems. Specifically, VL molecules with zincophilic functional groups can preferentially adsorb onto the Zn anode surface, reconstructing a electric double layer (EDL) and reducing HER occurrence. The adsorbed VL further undergoes ring‐opening reactions in response to the increased H^+^ concentration and mitigates the concentration polarization due to Zn^2+^ depletion in the SEI layer. Furthermore, the in situ polymerized protective layer (derived from VL) exhibits exceptionally high mechanical strength, significantly suppressing SEI fracture caused by volumetric strain and the subsequent side reactions. To further verify the advantage of VL as a co‐solvent, we have evaluated the electrochemical performance of Zn–I_2_ full batteries. The results demonstrate that VL effectively suppresses iodine dissolution, thereby significantly mitigating the shuttle effect of polyiodides. Benefiting from these synergistic effects, the symmetrical battery demonstrates stable cycling performance for over 4000 cycles at 40 mA cm^−2^ and 1 mAh cm^−2^. The Zn–I_2_ full battery demonstrates excellent rate capability and achieves 26 500 stable cycles at 50 C with a capacity retention rate of 86.4%. This co‐solvent and pH buffering strategy offers valuable insights into long‐life aqueous Zn–I_2_ batteries and lays a solid foundation for the design and optimization of high‐performance electrolytes.

## Results and Discussion

**Figure 1:**
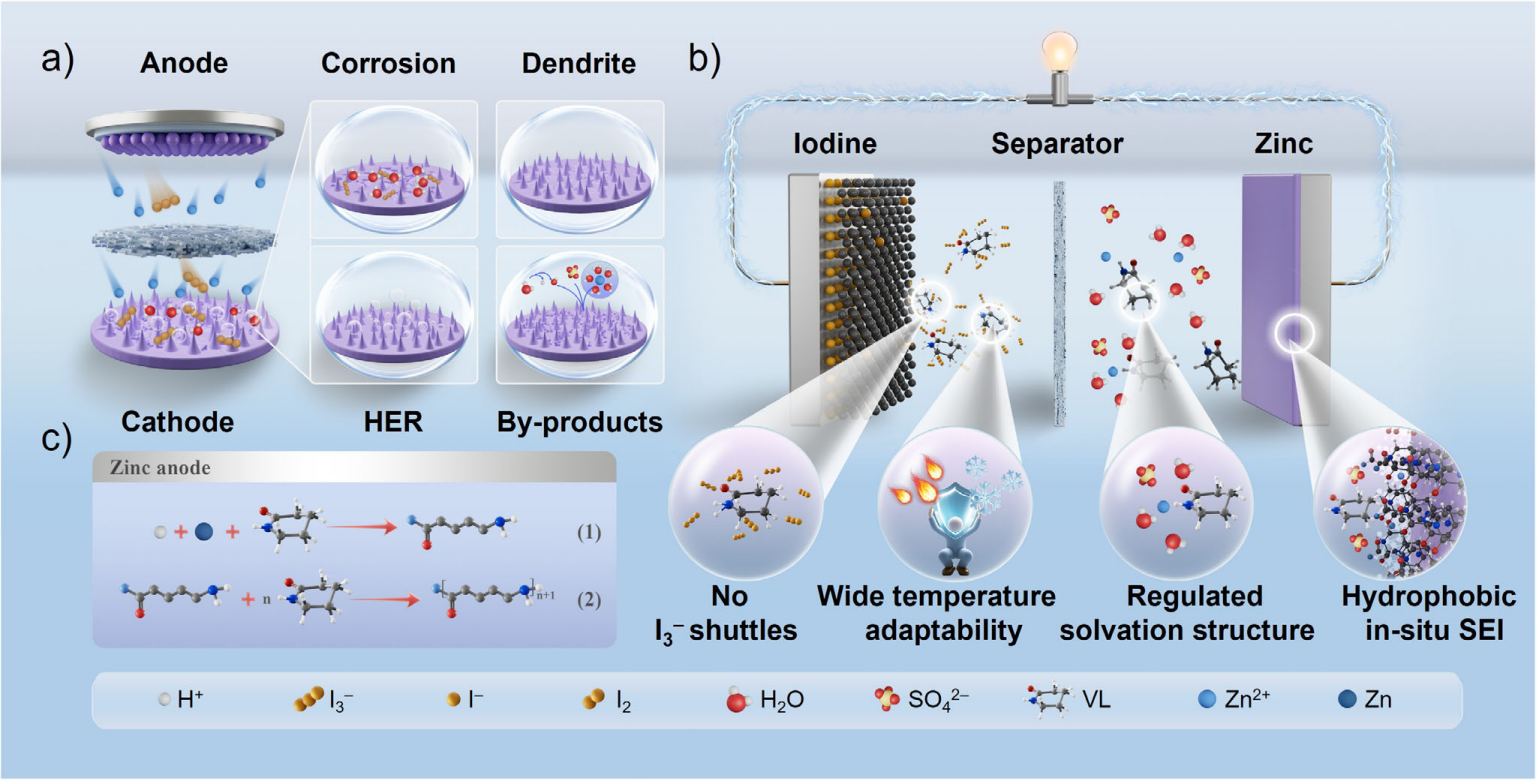
a) Schematic illustration of side reactions and dendrite growth in BE. b) Schematic illustration of the synergistic optimization strategy with iodine cathode, electrolyte, and in situ anode protection layer. c) Mechanism diagram of VL polymerization.

Zn–I_2_ batteries using the conventional bare ZnSO_4_ electrolytes (BE) often suffer from Zn anode corrosion, dendrite growth, and HER. And the dissolution and diffusion of polyiodides lead to the loss of active material and further aggravate anode corrosion (I_3_^−^ + 2e^−^ → 3I^−^, Zn – 2e^−^ → Zn^2+^) (Figure 1a).^[^ ^32^, ^33^ ^]^ To address these challenges, this study innovatively introduces VL, a highly soluble co‐solvent with a high donor number, into the aqueous Zn–I_2_ battery electrolyte. VL modulates the solvation structure of hydrated Zn^2+^, reducing the content of active water. Additionally, the zincophilic nature of VL enables its preferential adsorption onto the Zn anode surface, forming a H_2_O‐ deficient EDL. More importantly, the amine groups in VL interact with H^+^ and iodine species, water‐ dynamically regulating the local pH and effectively suppressing the polyiodide shuttle effect. Specifically, VL undergoes ring‐opening polymerization under specific chemical conditions, in situ forming a mechanically robust polymeric protective layer on the electrode surface. VL‐containing electrolyte (VL‐BE) also suppresses the transformation of polyiodides by increasing the enthalpy change of I_3_^−^ conversion, effectively preventing Zn anode corrosion (Figure 1b).

To further elucidate the working mechanism of VL, the reaction pathways are depicted in Figure 1c. Zn metal interacts with carbonyl oxygen atoms in the presence of H^+^, facilitating charge transfer to amine N atoms and generating positive charges. Subsequently, the amine N, acting as a nucleophile along with excess electrons, attacks the carbonyl oxygen of VL monomers, initiating a continuous ring‐opening polymerization reaction. This process not only stabilizes the local pH but also promotes the formation of a stable SEI protective layer. Moreover, the organic/inorganic interface facilitates Zn^2+^ desolvation, thereby reducing side reactions and regulating uniform Zn deposition. Through this synergistic modulation mechanism, a Zn anode with high thermodynamic stability and reversible plating/stripping behavior is expected to be achieved.

**Figure 2:**
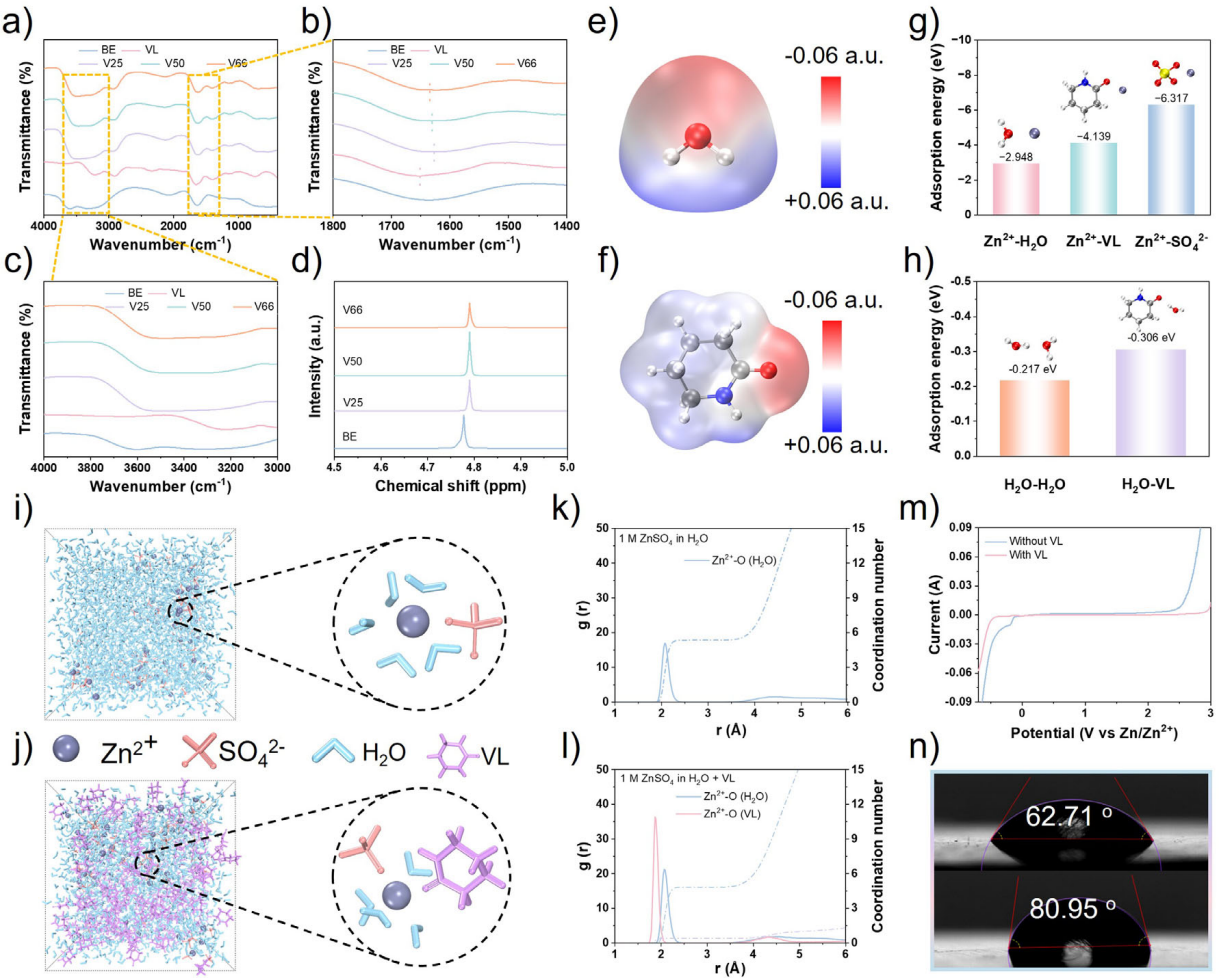
a–c) FT‐IR spectra of various electrolytes. d) NMR spectroscopy of various electrolytes. Electrostatic potential mapping of e) H_2_O and f) VL. g) Computed adsorption energies for H_2_O, VL, and SO_4_^2−^ with Zn^2+^. h) Computed adsorption energies for H_2_O and VL with H_2_O. i) and j) MD simulation images and k) and l) radial distribution function curves of BE and VL‐BE, respectively. m) LSV curves of different electrolytes tested on Ti electrodes. n) The contact angle of BE and VL‐BE.

VL, with its high solubility and high donor number, can be miscible with water.^[^ ^34^ ^]^ By mixing VL with ZnSO_4_ solution at different mass ratios of 1:3, 1:1, and 2:1, a series of solutions were obtained, named V25, V50, and V66, respectively. To verify the role of VL in regulating the coordination structure of hydrated Zn^2+^, Fourier‐transform infrared (FT‐IR) spectroscopy was used to analyze the changes in different functional groups in the solution. The results show that compared to pure VL, the C═O stretching vibration peak experiences a redshift. This can be attributed to the metal–oxygen coordination between Zn^2+^ and the carbonyl group in VL, confirming the strong affinity between Zn^2+^ and VL (Figure 2a,b).^[^ ^23^, ^35^ ^]^ In addition, compared to BE, the O─H stretching vibration peak also presents significant shift (Figure 2c), indicating that VL effectively disrupts the hydrogen bonding network of water molecules, thereby reducing the activity of free water.^[^ ^36^ ^]^ This can help to suppress the HER and corrosion reactions. Similar result is also confirmed by nuclear magnetic resonance (NMR). The ^1^H peak of BE is located at 4.768 ppm. As the VL concentration increased, the ^1^H peak gradually shifts to 4.791 ppm, further suggesting that VL can replace some water molecules and reconstruct the solvation shell of hydrated Zn^2+^ (Figure 2d).^[^ ^36^ ^]^ To theoretically examine the effect of VL on improving the solvation shell of hydrated Zn^2+^, the electrostatic potentials (ESP) of water and VL molecules were calculated (Figures 2e,f, S1, and S2). It was found that ESP_min_ of VL is lower than that of water molecules, indicating that VL can replace some water molecules in the Zn^2+^ solvation sheath,^[^ ^37^, ^38^ ^]^ which is consistent with the FT‐IR and NMR results.

Density functional theory (DFT) calculations and molecular dynamics (MD) simulations were used to visually analyze the first solvation shell of hydrated Zn^2+^ in the BE and VL‐BE systems. First, the interactions between Zn^2+^, SO_4_^2−^, H_2_O, and VL molecules were investigated (Figure 2g). The results show that the adsorption behavior of Zn^2+^‐VL is significantly stronger than that of Zn^2+^–H_2_O, indicating that Zn^2+^ prefers coordination with VL over water molecules. When comparing the adsorption energies of H_2_O–H_2_O and H_2_O–VL, it is found that H_2_O also tends to form intermolecular interactions with VL (Figure 2h). These results confirm that VL can disrupt the original hydrogen bonding network of H_2_O and replace some H_2_O in the first solvation structure of Zn^2+^. To further quantify the changes in water content within the solvation shell, MD simulations were conducted to analyze the solvation structure of Zn^2+^ in different systems (Figure 2i,j). Radial distribution function (RDF) and coordination number results show that the distance between Zn^2+^ and O of VL is obviously closer than that of H_2_O in the first solvation shell of Zn^2+^, which proves that the introduction of high donor VL can coordinate with Zn^2+^ to improve the solvated shell of hydrated Zn^2+^ (Figure 2k,l). Compared to the BE, the number of H_2_O molecules in the first solvation shell of Zn^2+^ in the VL‐BE system is significantly reduced. This further confirms that the introduction of VL optimizes the solvation structure of Zn^2+^, improves Zn^2+^ transport efficiency, and effectively suppresses HER during Zn deposition.^[^ ^39^ ^]^ These results are further validated by linear sweep voltammetry (LSV) measurements. As shown in Figure 2m, the VL‐containing electrolyte exhibits a significantly higher hydrogen evolution overpotential compared to the VL‐free counterpart, confirming the synergistic modulation effect of VL in stabilizing the Zn anode. In addition, the incorporation of VL leads to a notable expansion of the electrochemical stability window.

To determine the optimal VL concentration, Zn||Zn symmetric cells were assembled using the different mass ratios (V25, V50, and V66) of the mixed solutions as electrolytes. Electrochemical results showed that at a plating capacity of 1 mAh cm^−2^ and a current density of 20 mA cm^−2^, the symmetric cell using the V25 electrolyte exhibits the longest cycle life, exceeding 1400 cycles (Figure S3). This result may be related to the viscosity of the electrolyte and the Zn^2+^ conductivity. Excessive VL can increase the electrolyte viscosity and decrease Zn^2+^ conductivity, which would negatively affect battery performance.^[^ ^40^ ^]^ Based on these results, V25 was identified as the optimal ratio, and all subsequent experiments were performed using this electrolyte. The mean squared displacement (MSD) (Figure S4) and electrochemical impedance spectroscopy (EIS) (Figure S5) results support this estimation. Additionally, the initial deposition voltage curves measured under different electrolyte conditions also verified this result. In the BE system, the nucleation overpotential of Zn is lower than that in the VL‐BE system (Figure S6). Interfacial wettability plays a key role in Zn^2+^ transfer and the suppression of water decomposition. The wettability of the electrolyte before and after VL addition was evaluated by contact angle measurements. Compared to BE, the contact angle of VL‐BE drops sharply from 80.95° to 62.71°, demonstrating the excellent wettability of VL‐BE on the pristine electrode (Figure 2n).^[^ ^41^ ^]^

**Figure 3:**
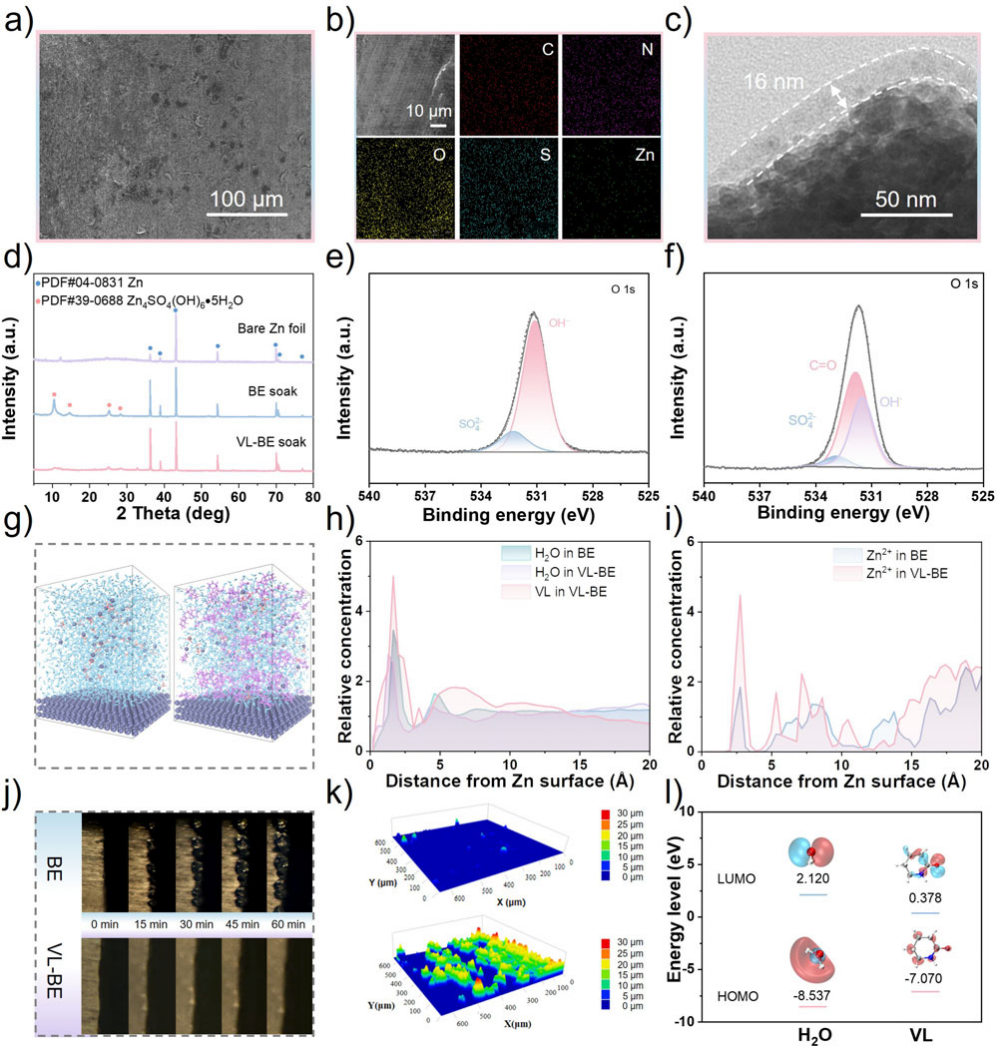
a) SEM image of Zn foil immersed in VL‐BE. b) Elemental distribution analysis of Zn foil immersed in VL‐BE. c) Transmission electron microscopy (TEM) image of the Zn electrode with the VL‐derived SEI protective layer. d) XRD pattern of the Zn foil immersed in various electrolytes. e) and f) XPS spectra of Zn surface immersed in BE and VL‐BE, respectively. g) “Snapshots” of the concentration distribution of H_2_O and VL on the Zn anode surface in MD simulations. h) Relative concentration of VL and H_2_O on the Zn anode surface. i) Relative concentration of Zn^2+^ on the Zn anode surface. j) In situ optical microscopy visualization of Zn plating behavior on Zn foil. k) 3D laser optical images of the Zn anodes in BE and VL‐BE. l) HOMO and LUMO energy levels for H_2_O and VL via DFT.

The protective effect of VL on the Zn anode can be determined by studying whether VL‐BE can enhance the stability of the Zn anode. Zn foils were immersed in VL‐BE and BE for 7 days, and their surface morphology was observed. Scanning electron microscope (SEM) images clearly show significant differences between the two: Zn foil immersed in BE exhibits a large amount of corrosion products on its surface (Figure S7), while the Zn foil in VL‐BE remains smooth and flat, with no obvious signs of corrosion (Figure 3a). Elemental distribution analysis shows that the Zn foil immersed in BE has uneven distributions of O, S, and Zn elements, with O and S elements notably accumulating in the corrosion product areas (Figure S8). In contrast, the Zn foil in the VL‐BE system shows uniform distributions of C, N, O, S, and Zn elements (Figure 3b), indicating that VL molecules form a stable protective layer on the Zn foil surface. As shown in Figure 3c, the thickness of the protective layer is approximately 16 nm. Further analysis using X‐ray diffraction (XRD) confirmed the composition of the material on the surface of the Zn foil after immersion.^[^ ^42^ ^]^ The XRD results show that, compared to bare Zn foil and Zn foil in the VL‐BE system, the Zn foil in the BE system has a prominent formation of Zn_4_SO_4_(OH)_6_·5H_2_O by‐product on its surface (Figure 3d). Additionally, to more accurately assess the impact of different electrolytes on Zn anode corrosion kinetics, Tafel tests were performed (Figure S9). The fitted linear polarization data indicates that, compared to BE, the electrode using VL‐BE is more resistant to corrosion. This corrosion resistance can be attributed to the formation of a stable SEI layer on the Zn electrode surface.

DFT calculations were employed to investigate the interactions between VL and H_2_O molecules on the Zn foil surface, aiming to gain deeper insight into how VL forms a protective layer on the Zn anode. As shown in Figures S10–S12, the adsorption energy of VL‐Zn is −0.696 eV, significantly higher than that of H_2_O‐Zn (−0.186 eV), proving that VL preferentially adsorbs onto the Zn surface and effectively repels H_2_O molecules, thereby forming a protective layer. X‐ray photoelectron spectroscopy (XPS) was used to confirm the nature of the interactions between VL and the Zn anode surface (chemical or physical adsorption) (Figures 3e,f and S13).^[^ ^43^ ^]^ As shown in Figure 3e, the O 1s spectrum of the Zn foil immersed in BE contains two peaks from SO_4_^2−^ and OH^−^, which can be attributed to the chemical corrosion from the electrolyte. In contrast, the Zn foil immersed in VL‐BE shows the presence of a clear C═O peak in addition to SO_4_^2−^ and OH^−^ (Figure 3f), confirming that VL physically adsorbs onto the Zn anode surface, enhancing its corrosion resistance.

The protective effect of VL on the Zn anode is also reflected in its significant ability to suppress dendrite growth. The influence of different electrolytes on Zn nucleation behavior was studied using chronoamperometry (CA) (Figure S14a). In the BE system, the current of Zn continuously increases within 200 s, indicating that an uncontrolled 2D diffusion process led to the ongoing side reactions, which in turn exacerbated dendrite growth.^[^ ^44^ ^]^ In contrast, in the VL‐BE system, Zn rapidly transitions from 2D diffusion to 3D diffusion after a brief period, suggesting that the VL‐BE system promotes uniform Zn deposition by increasing the number of nucleation sites. This result can be further validated by DFT calculations (Figures S14b,c, S15, and S16). In the presence of VL, the adsorption energy of Zn^2+^ is higher than on the bare Zn electrode surface, indicating that VL can increase the diffusion energy barrier of Zn^2+^, thus effectively suppressing its disordered diffusion. The main cause of Zn dendrite formation is that Zn^2+^ is more likely to nucleate in regions with lower surface energy, leading to preferential growth at the tips, which induces dendrite formation.^[^ ^35^, ^42^ ^]^ These results demonstrate that VL‐based electrolytes can effectively suppress Zn dendrite growth and promote uniform Zn deposition.

MD simulations were used to analyze the distribution of water molecules and Zn^2+^ on the Zn anode surface under different electrolyte systems to investigate the role of VL in regulating the electrolyte/electrode interface (Figures 3g‐i and S17). The results show that in the VL‐BE system, VL preferentially adsorbs on the Zn anode surface, forming a spatially effective SEI protective layer, which can prevent the direct contact between H_2_O molecules and the electrode. With H_2_O molecules in the solution being partially replaced by VL, the combined effect of both leads to a significant reduction in the number of water molecules on the electrode surface, thereby suppressing HER. The protective effect of VL on the Zn anode can also be achieved through the adsorption of H^+^ on the Zn anode surface, where in situ ring‐opening occurs. This can be proved by the change in pH of the electrolyte with increasing cycle numbers in different electrolytes. As shown in Figure S18, with the increase in battery cycling time, the introduction of VL significantly slows down the increase in pH, and after 100 cycles, the final pH value is lower. This can be attributed to the VL molecules adsorbed on the Zn anode surface, which are capable of capturing H⁺ and undergoing in situ ring‐opening, thereby regulating the local pH and stabilizing the overall pH of the electrolyte. Moreover, the self‐assembled SEI protective layer formed by the ring‐opening polymerization significantly alleviated the issue of increasing OH^−^ concentration due to HER, thus preventing the formation of Zn_4_SO_4_(OH)_6_·5H_2_O and the corrosion of the Zn anode. Additionally, the in situ ring‐opening VL binds to Zn^2+^ and undergoes in situ polymerization with other VL molecules, forming a high mechanical strength SEI layer, which significantly mitigates the polarization problem caused by concentration differences; thereby, the cycling performance of the cell can be improved.

As shown in Figure 3j, the deposition process of Zn in BE and VL‐BE was monitored using optical microscopy, providing a more intuitive demonstration of the protective effect on the Zn anode. After 15 min, dendrite growth due to uneven deposition is observed on the Zn foil surface in BE. These disordered Zn dendrites increase the surface area of the Zn foil, thereby exacerbating the HER. In contrast, the Zn foil in VL‐BE maintains a smooth surface throughout the electroplating process, even after 60 min of deposition, without any protrusions. The SEM results further confirmed these observations. SEM was used to characterize the surface morphology of the Zn anode in different electrolytes at different electroplating capacities (Figures S19 and S20). At a plating capacity of 1 mAh cm^−2^, the surface of the Zn foil in VL‐BE remains intact, whereas the surface in BE exhibits noticeable dendrite growth. When the capacity is increased to 5 mAh cm^−2^, the surface morphology in VL‐BE still maintains this trend. These results indicate that VL induces uniform deposition of Zn on the Zn foil surface. The height measurements of the electrode surface further support this conclusion. The 3D laser optical images clearly show that the Zn foil in VL‐BE has a higher surface flatness, whereas the surface in BE is rougher (Figure 3k), further proving that VL effectively suppresses Zn dendrite growth and enhances the stability of the Zn anode. To further investigate the electron transfer behavior of VL and H_2_O molecules at the Zn interface, molecular orbital (MO) analysis was performed. The energy gap between the lowest unoccupied molecular orbital (LUMO) and the highest occupied molecular orbital (HOMO) determines the stability of the electrochemical window. The calculation results show that, compared to H_2_O, VL has a lower energy gap and a lower LUMO energy level, indicating that VL is more likely to act as an electron acceptor, forming a stable SEI layer on the Zn anode surface (Figure 3l).^[^ ^38^ ^]^ The Gibbs free energy calculation result further supports this result. The interaction between Zn metal and the carbonyl oxygen atom of the VL molecule, facilitated by H⁺, triggers the ring‐opening polymerization reaction of VL, with a Gibbs free energy of −82.45 kcal mol^−1^, suggesting that this process is spontaneous. The dual evidence from both experiments and theoretical calculations shows that VL not only regulates the solvation shell of Zn^2+^ but also preferentially adsorbs on the Zn metal surface, disrupting the hydrogen bonding network of H_2_O molecules, thus constructing a protective layer. Moreover, the ring‐opening polymerization of VL effectively regulates the local pH, suppressing HER corrosion and the formation of “dead Zn.”

**Figure 4:**
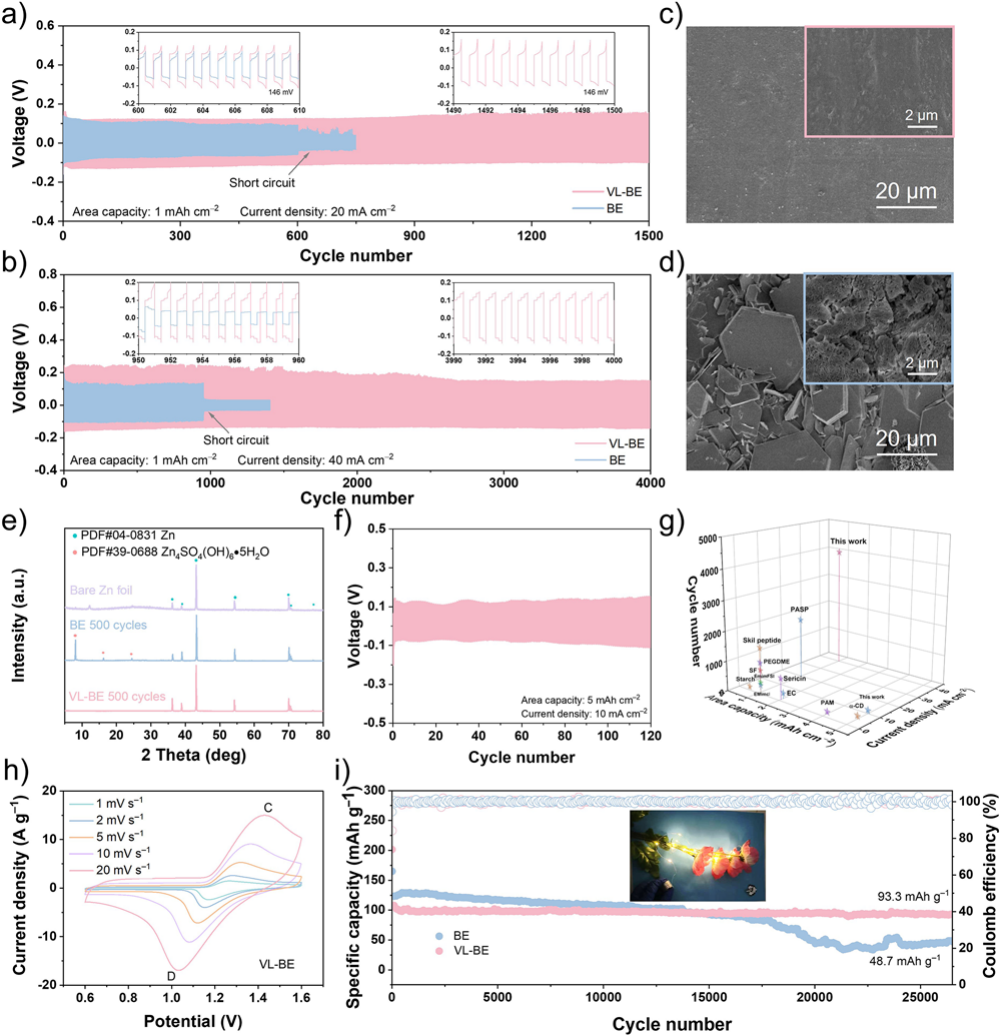
a) and b) Voltage profiles for Zn symmetric batteries tested in BE and VL‐BE with current density of (a) 20 mA cm^−2^ and (b) 40 mA cm^−2^ and capacity of 1 mAh cm^−2^. c) and d) SEM images of Zn anode in (c) VL‐BE and (d) BE after 100 cycles. e) XRD pattern of Zn anode after 500 cycles. f) Voltage profiles for Zn symmetric battery tested in VL‐BE with current density of 10 mA cm^−2^ and capacity of 5 mAh cm^−2^. g) Comparison of the cycle life of VL‐BE with reported electrolytes under different capacities and current densities. h) CV curves of VL‐BE determined from 0.6 V to 1.6 V at various scan rates. i) Cycle stability for Zn||I_2_ full batteries using BE and VL‐BE at 50 C. The inset is digital photograph of battery as energy source to power LED indicator.

The protective effect of VL on the Zn anode can be evaluated by the long‐term cycling stability of symmetric batteries. As shown in Figure 4a, under a current density of 20 mA cm^−2^ and a plating/stripping capacity of 1 mAh cm^−2^, the symmetric cell with VL‐BE electrolyte can cycle stably for more than 1500 cycles. The magnified voltage profile shows that no short circuit occurs, and the overpotential remains around 146 mV. In contrast, the symmetric cell using BE exhibits a sharp drop in polarization voltage after 600 cycles, indicating severe dendrite growth and corrosion issues. When the current density is further increased to 40 mA cm^−2^, the VL‐BE symmetric battery still maintains stable cycling for over 4000 cycles (Figure 4b). Moreover, cycling tests conducted at lower current densities (1, 2, and 10 mA cm^−2^) further validate the exceptional stability of cells employing the VL‐BE electrolyte (Figures S21‐S23). To further verify the superiority of VL as an electrolyte solvent component, comparisons with previous literature show that the cells using VL‐BE electrolyte demonstrate outstanding electrochemical performance (Figure 4g).^[^ ^45^, ^46^, ^47^, ^48^, ^49^, ^50^, ^51^, ^52^, ^53^, ^54^, ^55^ ^]^ Rate performance tests were conducted to further confirm the commercial application potential of the VL‐BE electrolyte. As shown in Figure S24, the symmetric cell with VL‐BE electrolyte maintains excellent cycling stability under different current densities of 1, 3, 5, and 10 mA cm^−2^, proving its superior rate capability. To directly validate the suppression effect on dendrite growth and corrosion of VL, Zn foils cycled in different electrolyte environments were analyzed by SEM. As shown in Figures 4c,d and S25, after 100 cycles in BE electrolyte, the Zn foil surface exhibits significantly increased roughness along with disordered dendrite growth. In contrast, the Zn foil in VL‐BE electrolyte maintains a smooth and uniform surface. XRD analysis further confirms the anticorrosion ability of VL for the Zn anode. The Zn foil cycled in BE shows the formation of Zn_4_SO_4_(OH)_6_·5H_2_O byproduct, whereas no byproduct phase is detected on the Zn foil cycled in VL‐BE electrolyte (Figure 4e).

To further demonstrate the reliability of VL‐BE as a commercial electrolyte, the plating/stripping capacity was increased to 5 mAh cm^−2^. As shown in Figures 4f and S26, at the current density of 10 mA cm^−2^, the symmetric cell using the VL‐BE electrolyte maintains stable cycling for 120 cycles with an overpotential of only 112 mV. Even at higher current densities and area capacities, the VL‐BE electrolyte maintains superior cycling performance compared to the BE system (Figure S27). SEM observations further verify that the VL‐BE electrolyte effectively inhibits dendrite formation, which in turn extends the cycling life of the cell (Figure S28). Coulombic efficiency (CE) is a key parameter for evaluating the reversibility of Zn plating/stripping processes. As shown in Figure S29, the cell with BE exhibits an initial CE of 88.47% at a current density of 20 mA cm^−2^, but the CE drops after six cycles, likely due to dendritic Zn growth and associated side reactions. In contrast, the cell employing the VL‐BE shows a higher initial CE of 91.78%, and then rapidly increased to 99.77%. These results demonstrate that VL effectively suppresses parasitic reactions and improves the reversibility of Zn plating/stripping. Additionally, long‐cycle performance tests under low VL concentrations (0.05 mg mL^−1^) show that even at a plating/stripping capacity of 1 mAh cm^−2^ and current densities of 20 and 40 mA cm^−2^, the VL‐BE electrolyte still exhibits significantly enhanced cycling stability (Figure S30). This trend is further confirmed by rate test, fully highlighting the great advantages of VL‐BE electrolyte in Zn–I_2_ batteries.

This remarkable improvement can be attributed to the ability of VL to optimize the solvation structure of Zn^2+^, disrupt the hydrogen bonding network of H_2_O molecules, dynamically neutralize H^+^, and form a mechanically robust, Zn^2+^‐rich SEI layer on the Zn anode surface, effectively suppressing side reactions. To analyze the chemical composition and the stability of the SEI layer formed on the Zn anode in the VL‐BE electrolyte, XPS was used to analyze the SEI layer at different depths before and after cycling (Figure S31). In the pre‐cycling C 1s spectra, peaks corresponding to C═O, C─O, and C─C are observed at different depths (Figure S31a).^[^ ^56^ ^]^ As the etching depth increased, these signal peaks remained in the spectra, indicating that VL undergoes ring‐opening and polymerization reactions, forming a SEI protective layer that adheres to the Zn anode surface. Moreover, in the post‐cycling C 1s spectra, these characteristic peaks remain stable (Figure S31b). This proves that the SEI layer on the Zn anode surface remains compositionally stable during cycling, highlighting the promising application potential of VL as a pH buffer in electrolyte.

In practical applications, cell performance is inevitably affected by operating temperature, especially in extreme environments where overall performance can significantly decline. At subzero temperatures, the electrolyte undergoes irreversible phase transitions, and its viscosity increases, limiting Zn^2+^ transport. In high‐temperature environments, the surface migration energy barrier decreases, accelerating ion diffusion and nucleation processes, which leads to increased disorder in Zn deposition. Additionally, factors such as increased interfacial voids and water evaporation further impact battery stability and cycle life.^[^ ^35^, ^57^ ^]^ The key to achieving a wide‐temperature‐tolerant aqueous Zn–I_2_ battery lies in suppressing water activity. Potential solutions include reconstructing the hydrogen bonding network and eliminating solvated water. Previous experimental results have demonstrated that VL‐BE possesses these characteristics, suggesting its potential for enabling wide‐temperature‐adaptive Zn–I_2_ batteries. First, we investigated the effect of VL on the low‐temperature phase transition of the electrolyte (Figure S32). At −10 °C, the BE system solidifies, whereas the VL‐BE system remains liquid. To further evaluate its low‐temperature performance, symmetric cells were assembled and assessed for their long‐cycle stability at −5 °C (Figure S33). Under a current density of 10 mA cm^−2^ and a plating/stripping capacity of 1 mAh cm^−2^, the VL‐BE system achieves stable cycling for 450 cycles with an overpotential of only 170 mV. Furthermore, at an elevated temperature of 50 °C (Figure S34), the BE system suffers from dendrite‐induced short circuits after only 49 cycles, whereas the VL‐BE system remains stable for 550 cycles while maintaining a low overpotential (160 mV). These results further validate the superior performance of VL‐BE under extreme temperature conditions, highlighting its outstanding potential for practical applications.

**Figure 5:**
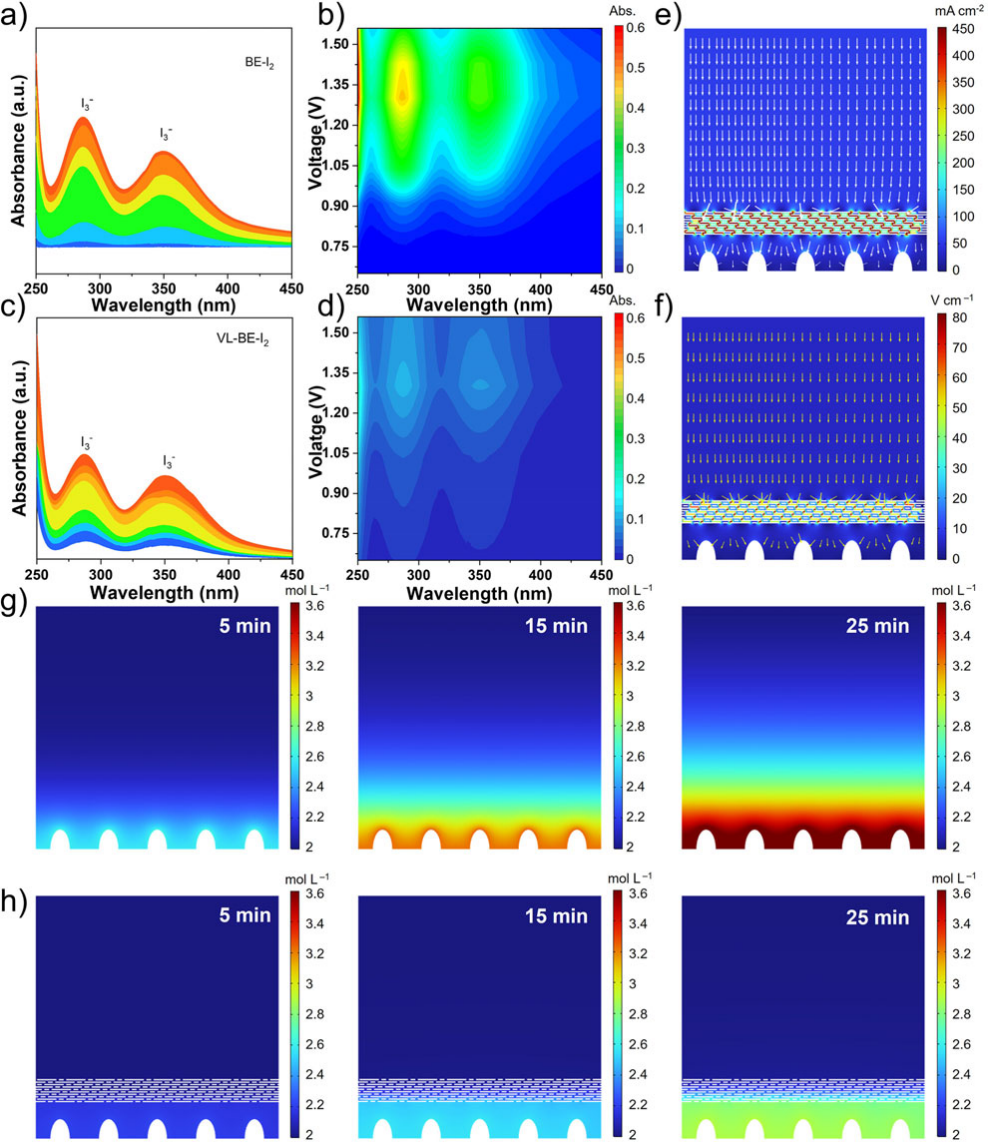
In situ UV spectra of a) and b) BE and c) and d) VL‐BE. e) Simulation of current density distribution for the Zn anodes in VL‐BE. f) Models for the relative intensity distributions of localized electric fields for the Zn anodes in VL‐BE during Zn nucleation. Concentration field simulation at the interface of Zn anodes in g) BE and h) VL‐BE after constant diffusion time.

Further investigation of the full battery electrochemical performance was conducted to verify that VL‐BE can suppress the polyiodide shuttle effect. Cyclic voltammetry (CV) tests were conducted to evaluate the redox kinetics of BE and VL‐BE electrolytes. As depicted in Figures 4h, S35, and S36, VL‐BE exhibits lower electrochemical polarization across different scan rates, suggesting superior redox kinetics during charge and discharge processes. A comparison of the CV curves further highlights that VL‐BE possesses higher redox reversibility (Figure S37). Furthermore, the charge and discharge cycling performance of both electrolytes was examined at 50 C in Zn||I_2_ full batteries (Figure 4i). VL‐BE maintains excellent stability over 26 500 cycles, retaining a capacity of 93.3 mAh g^−1^ with a capacity retention of 86.4%. In contrast, although BE initially exhibits a higher capacity, it rapidly decayed within a relatively short cycle life, with the capacity dropping to only 48.7 mAh g^−1^ after 26 500 cycles. The outstanding electrochemical performance of VL‐BE can be attributed to two key factors. First, for the iodine cathode, VL exhibits an adsorption effect on I_2_, effectively mitigating iodine dissolution and shuttle effect. This result can be confirmed by the in situ UV results. As shown in Figure 5a–d, compared to BE, the solubility of I_3_^−^ in VL‐BE is significantly lower, demonstrating that VL can effectively inhibit the shuttle effect of polyiodides. Second, the ring‐opened VL also interacts with Zn^2+^ and undergoes in situ polymerization, forming a high mechanical strength SEI layer enriched with Zn^2+^. This layer reduces concentration polarization, alleviates side reactions between Zn and the electrolyte, and accelerates the catalytic conversion of polyiodides. Finally, simulations were conducted to analyze the evolution of current density and electric field intensity during Zn deposition in both VL‐BE and BE systems. Notably, randomly distributed defects exhibit higher current density and electric field intensity than other areas of the Zn foil, leading to uneven dendrite growth (Figure S38). However, the in situ formed protective layer in the VL‐BE system effectively homogenizes current density and electric field distribution (Figure 5e,f). In addition, to further explore Zn plating/stripping behavior in different electrolytes, we simulated the Zn^2+^ concentration distribution on the Zn foil surface (Figure 5g,h). As the reaction proceeds, the Zn^2+^ concentration in the VL‐BE remains more uniform compared to that in BE, indicating that VL effectively facilitates spatially uniform Zn^2+^ distribution. Therefore, under high‐rate and high‐area‐capacity testing conditions, VL‐BE exhibits lower concentration polarization and superior rate performance compared to BE.

## Conclusion

In conclusion, a multifunctional electrolyte co‐solvent (VL) was designed to simultaneously stabilize the Zn anode and buffer the pH value at the anode/electrolyte interface. The ring‐opening of VL can dynamically neutralize H⁺ and combine with Zn^2+^, significantly increasing the Zn^2+^ concentration in the SEI layer, effectively alleviating the polarization issues caused by concentration gradients. The self‐polymerization of VL can form an SEI protective layer with high mechanical strength, ensuring a uniform distribution of Zn^2+^ and promoting the uniform deposition of Zn. Moreover, the developed electrolyte pH buffer can also minimize the shuttling effect of polyiodides and broaden the electrochemical window of aqueous Zn–I_2_ batteries. Benefitting from these synergistic effects, the full battery of Zn–I_2_ batteries with VL maintains nearly 100% CE after 26 500 cycles, with a capacity retention of 93.3 mAh g^−1^ and a capacity retention rate of 86.4% at 50 C. This multifunctional pH buffer design provides new insights into electrolytes, promoting the commercialization of aqueous Zn–I_2_ batteries.

Supporting Information includes 38 figures and 1 table.

## Conflict of Interests

The authors declare no conflict of interest.

## Supporting information



Supporting Information

## Data Availability

The data that support the findings of this study are available from the corresponding author upon reasonable request.
