# Supplementary material for: Synergistic Construction of In Situ Self‐Polymerized Interface and Localized pH Buffer Zone for High‐Performance Aqueous Zinc–Iodine Batteries
Source: Angew Chem Int Ed Engl. 2025 Aug 27;64(43):e202511490. doi: 10.1002/anie.202511490 (PMC12535400; doi:10.1002/anie.202511490)
Supplement: Supplementary file 1 — Supporting Information [file ANIE-64-e202511490-s001.docx]

Supporting Information
©Wiley-VCH 2021
69451 Weinheim, Germany

Synergistic Construction of In-situ Self-Polymerized Interface and Localized pH Buffer Zone for High-Performance Aqueous Zinc-iodine Batteries

Jiapei Li^[a]‡^, Zhiying Fang^[b]‡^, Hanjian Chen^[b]‡^, Kunlun Liu^[a]^, Yicai Pan^[a]^, Xiaoge Li^[b]^, Dewu Lin^[c]^ Nanyang Wang^[d]^*, Can Guo ^[e]^*, Cuiping Han^[f]^, Yagang Yao^[g]^, Pan Xue^[a, b]^*, & Guo Hong^[a, h]^*

[a] Department of Materials Science and Engineering & Center of Super-Diamond and Advanced Films (COSDAF), City University of Hong Kong, Hong Kong, 999077, P. R. China.

[b] School of Chemistry and Chemical Engineering, Yangzhou University, Yangzhou 225009, P. R. China.

[c] State Key Laboratory of Advanced Waterproof Materials, School of Advanced Materials, Peking University, Shenzhen Graduate School, Shenzhen 518055, P. R. China.

[d] NTI-NTU Corporate Laboratory, Nanyang Technological University, Singapore, 637662, Singapore.

[e] Department of Applied Biology and Chemical Technology, Faculty of Science, The Hong Kong Polytechnic University, Hong Kong 999077, P. R. China.

[f] Faculty of Materials Science and Energy Engineering, Shenzhen University of Advanced Technology, Shenzhen 518107, P. R. China.

[g] National Laboratory of Solid State Microstructures, College of Engineering and Applied Sciences, Jiangsu Key Laboratory of Artificial Functional Materials, Collaborative Innovation Center of Advanced Microstructures, Nanjing University, Nanjing 210023, P. R. China.

[h] The Shenzhen Research Institute, City University of Hong Kong, Shenzhen 518057, P. R. China.

^†^Jiapei Li, Zhiying Fang, Hanjian Chen contributed equally to this work.

*Correspondence and requests for materials should be addressed to N. W (email: nanyang.wang@ntu.edu.sg), C. G. ( email: canguo95@163.com), P. X. (email: panxue@yzu.edu.cn), and G. H. (email: guohong@cityu.edu.hk).

**Abstract:** Aqueous zinc-iodine (Zn-I_2_) batteries are promising for large-scale energy storage. However, their practical use is hindered by challenges such as Zn dendrite growth, hydrogen evolution reaction (HER), corrosion and polyiodide shuttle effect. In this study, valerolactam (VL) is employed as an organic pH buffer to address these issues. Theoretical and experimental results demonstrate that VL can regulate the electrolyte local pH while in-situ polymerizing on the electrode surface to form a mechanically stable solid electrolyte interphase (SEI) protection layer, effectively suppressing HER, corrosion and dendrite growth. Furthermore, the introduction of VL significantly regulates the solvation structure of Zn^2+^, and disrupts the inherent hydrogen bonding network, which enhances the electrochemical performance. As a result, a symmetric cell with VL-based electrolyte achieves impressive longevity under ultra-high current density (4000 cycles at 40 mA cm^–2^ and 1 mAh cm^–2^), 4.3 times higher than the counterpart in the conventional ZnSO_4_ electrolytes. Moreover, VL effectively suppresses polyiodide shuttle effect and improves electrochemical stability. Consequently, Zn-I_2_ full battery exhibits exceptional cycling stability, sustaining 26500 cycles with a high-capacity retention of 86.4%. Therefore, organic pH buffering engineering has been proved to be a promising strategy for achieving dendrite-free, shuttle-free Zn-I_2_ batteries.

DOI: 10.1002/anie.2025XXXXX

Characterization

The X-ray Diffraction (XRD) patterns of the samples were obtained using a Rigaku D/max 2200 PC diffractometer equipped with Cu Kα radiation. X-ray Photoelectron Spectroscopy (XPS) measurements were performed on a Thermo ESCALAB 250Xi spectrometer with monochromatic Al Kα radiation (hγ = 1486.6 eV). The surface morphology and structure were characterized using a field-emission scanning electron microscope (FE-SEM, Hitachi SU 8100). Transmission Electron Microscopy (TEM) image was captured with a JEM-2100F transmission electron microscope. The in-situ observation of Zn dendrite growth was conducted using an optical microscope (Leica DM750 M) equipped with a digital camera. The solvation structure of Zn^2+^ was analyzed using a nuclear magnetic resonance (^1^H NMR) spectrometer, with pure D_2_O replacing DI water as the solvent.

Experimental Procedures

**Electrochemical Measurements**

The electrochemical behavior of symmetric batteries was studied using a 2032-type coin-batterys, with either bare 1 M ZnSO_4_ (BE) or VL based ZnSO_4_ solution (VL-BE) as the electrolyte, and Zn foils as both the working and counter electrodes. Full batteries were assembled to assess the feasibility of the VL-BE. I_2_ electrodes were synthesized by mixing I_2_, super P, and polyvinylidene difluoride (PVDF) in a mass ratio of 7:2:1, using N-methyl-2-pyrrolidone (NMP) as the solvent. The active material (I_2_) loading was between 1.0 and 1.5 mg cm^−2^. Zn foil (thickness: 0.2 mm) was used as the anode. VL-BE was used as the electrolyte for testing the electrochemical performances of the full-batteries. The cycling performance of the batteries was evaluated using the LAND battery-testing system at various current densities. The CV with a voltage range of 0.6 to 1.6 V at various scan rates was tested via CHI 760E electrochemical workstation.

**Density functional theory (DFT) calculation**

For DFT calculation, the atomic charges and electrostatic potential (ESP) distributions were evaluated using the Gaussian 16 software package. The Becke, three-parameter, Lee-Yang-Parr (B3LYP) exchange-correlation functional was employed in conjunction with the 6-311++G (d, p) basis set.^[1]^ Adsorption energy calculations were conducted based on CASTEP packages, ^[2]^ the Generalized Gradient Approximation (GGA) and the Perdew-Burke-Ernzerhof (PBE) functional were applied. ^[3, 4]^ The plane-wave basis cutoff energy was set to 450 eV, and the 3×3×1 k-point setting and the Broyden-Fletcher-Goldfarb-Shannon (BFGS) algorithm was employed. ^[5]^ The geometry optimizations adhered to a stringent convergence criterion, with Hellmann-Feynman forces and the total energy difference are required to be smaller than 0.01 eV Å^−1^ and 1×10^-5^ eV atom^−1^, respectively. ^[6]^ The adsorption energy was computed from:

E_ad_=E_total_ -E_A_ -E_B_

**Molecular dynamics (MD) simulations**

For MD simulations, the Forcite module and the condensed-phase optimized molecular potentials for atomistic simulation studies (COMPASS) III force field were carried out. Electrostatic interactions were handled via the Ewald summation technique, while van der Waals interactions were calculated using an atom-based model. ^[7]^ Simulations employed with a two-phase equilibration approach: first, the systems were equilibrated under NPT conditions at 298 K for 200 ps to ensure the correct density and structural integrity of the amorphous battery, with a fixed time step of 1.0 fs. ^[8]^ Subsequently, the systems were stabilized under NVT conditions for 2000 ps, allowing for the relaxation and equilibrium of molecular dynamics. The final 200 ps of NVT results were used to analyze the redial distribution function (RDF) and relative concentration profiles. The mean square displacement (MSD) was used to investigate the effect of movement of Zn^2+^. The related equation is as follows:

$$\text{MSD=}\frac{\text{1}}{\text{N}}\sum_{\text{i=1}}^{\text{N}} {\text{[}\text{r}_{\text{i}}\left( \text{t} \right)\text{-}\text{r}_{\text{i}}\left( \text{0} \right)\text{]}}^{\text{2}}$$

where *r_i_ (t)* is the position of the center of mass of ion i at time t. For the electrolyte models, the compositions of H_2_O, VL, and ZnSO_4_ are provided in Table S1.

**Table S1.** The number of compositions in different electrolyte models.

| **Electrolyte** | **H_2_O** | **VL** | **ZnSO_4_** |
| --- | --- | --- | --- |
| H_2_O | 2775 | 0 | 50 |
| H_2_O + VL | 1850 | 168 | 50 |

**Finite element simulation**

Before the simulation, the geometric models for bare Zn and Zn@VL electrodes were initially designed. The length of two electrodes and the distance between them are both 10 μm. The protuberance on the surface of pristine Zn were set with a diameter of 0.8 μm and a height of 0.8 μm. In addition, the VL interface layer is represented by a rectangle of porous channels with a thickness of 1 μm, and the channels represent Zn^2+^ transport channels. However, this model can only establish an ideal system, and cannot fully reflect the real condition. And Finite element simulation based on the following partial differential equations ^[9, 10]^:

E=-∇V

$$\text{J}_{\text{i}}\text{=-}\text{D}_{\text{i}}\text{∇}\text{c}_{\text{i}}\text{-}\text{z}_{\text{i}}\text{μ}_{\text{m,i}}^{\text{∞}}\text{F}\text{c}_{\text{i}}\text{E}$$

$${\text{∂c}_{\text{i}}}/{\text{∂t=-}\text{∇}\text{∙}}\text{J}_{\text{i}}$$

where *E* is the electric field (V), *V* is the electric potential (V), *J_i_* is the flux vector (mol m^−2^ s^−1^), *D_i_* is the diffusion coefficient (m^2^ s^−1^), *c_i_* is the concentration (mol m^−3^), *z_i_* is the charge number, *μ∞ m,i* is the ion mobility (mol s kg^−1^) and *F* is the Faraday constant.

Results and Discussion


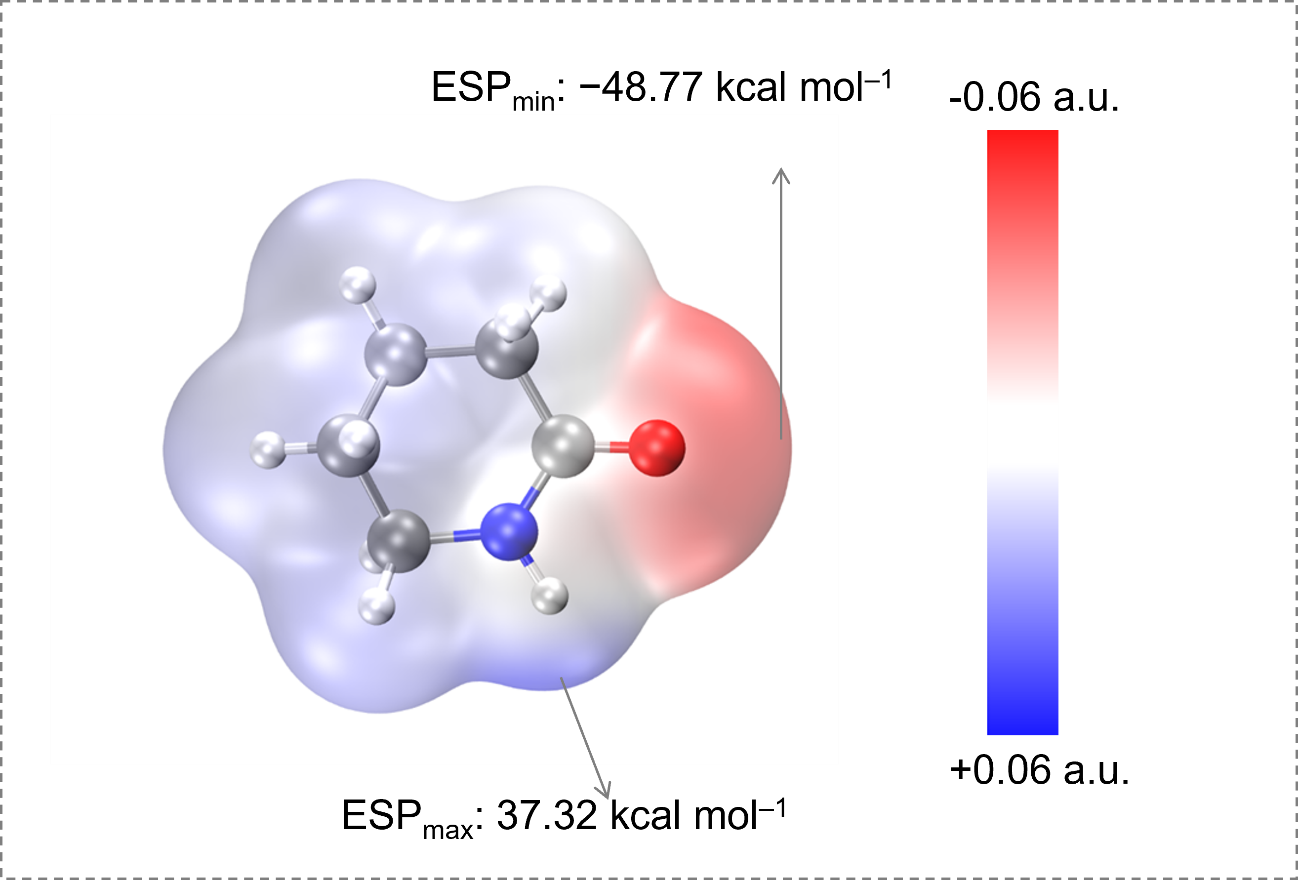


**Figure S1.** Electrostatic potential mapping of VL.


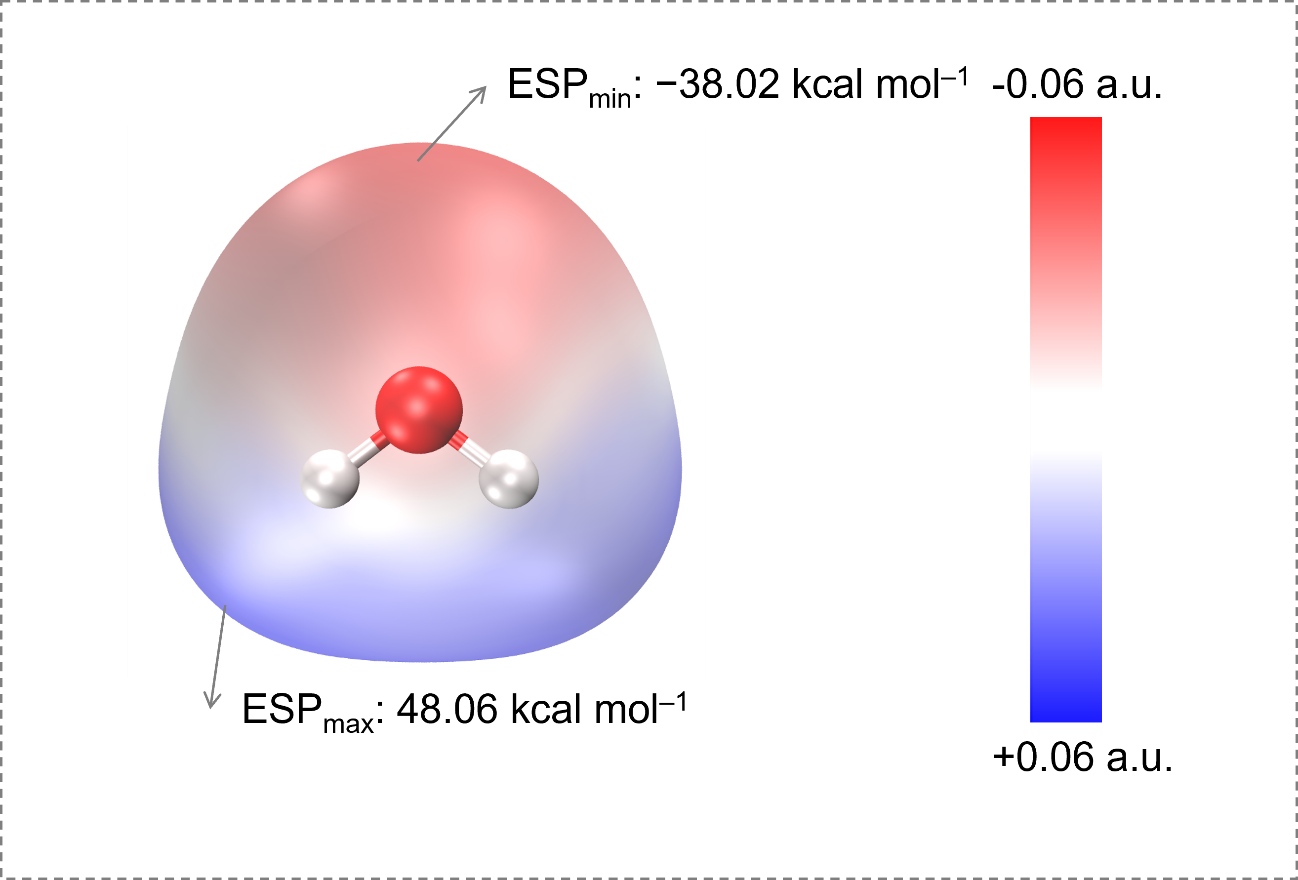


**Figure S2.** Electrostatic potential mapping of H_2_O.


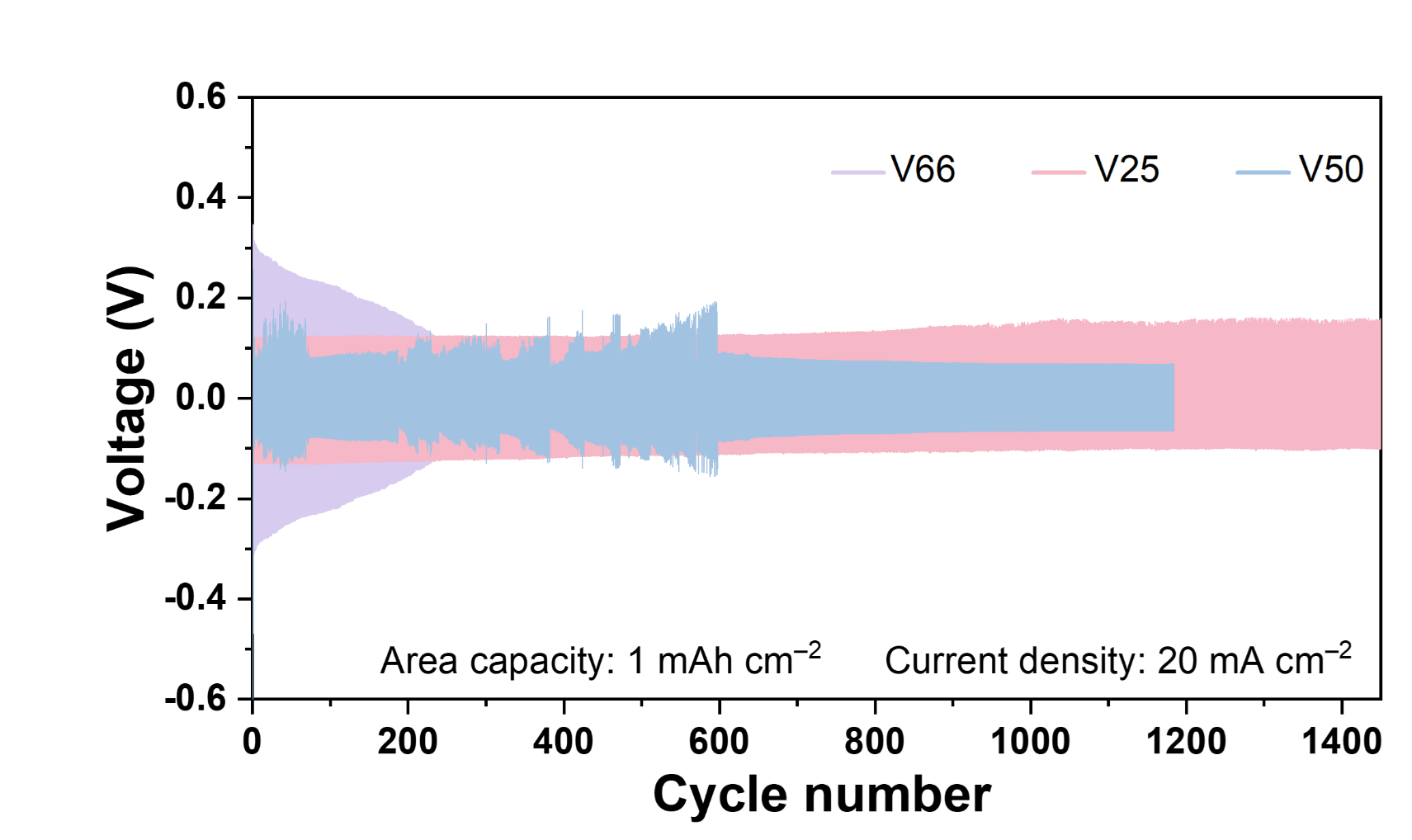


**Figure S3.** Voltage profiles for Zn symmetric batteries tested in various VL-BE electrolytes with current density of 20 mA cm^−2^ and capacity of 1 mAh cm^−2^.


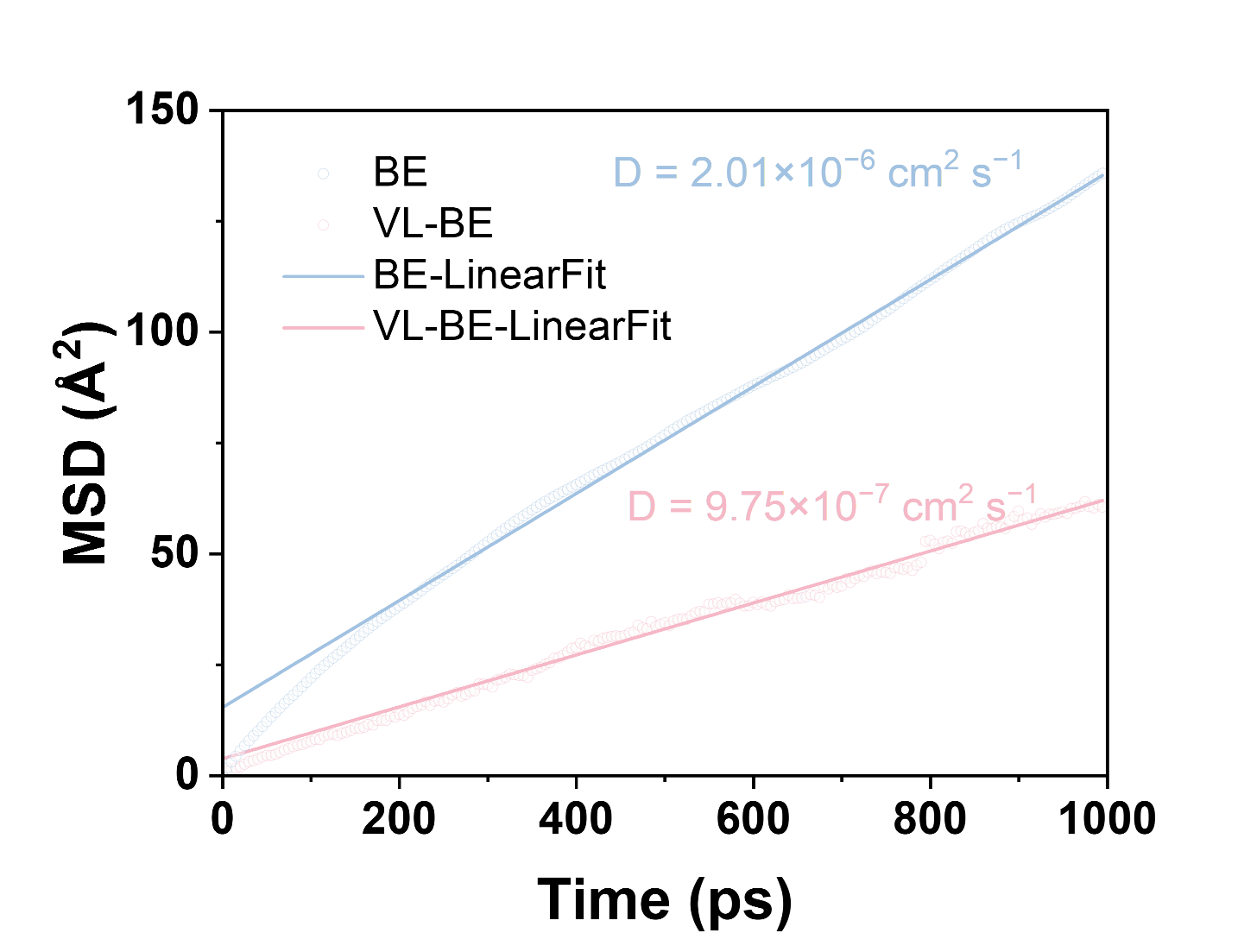


**Figure S4.** The mean squared displacement (MSD) results of BE and VL-BE.

**
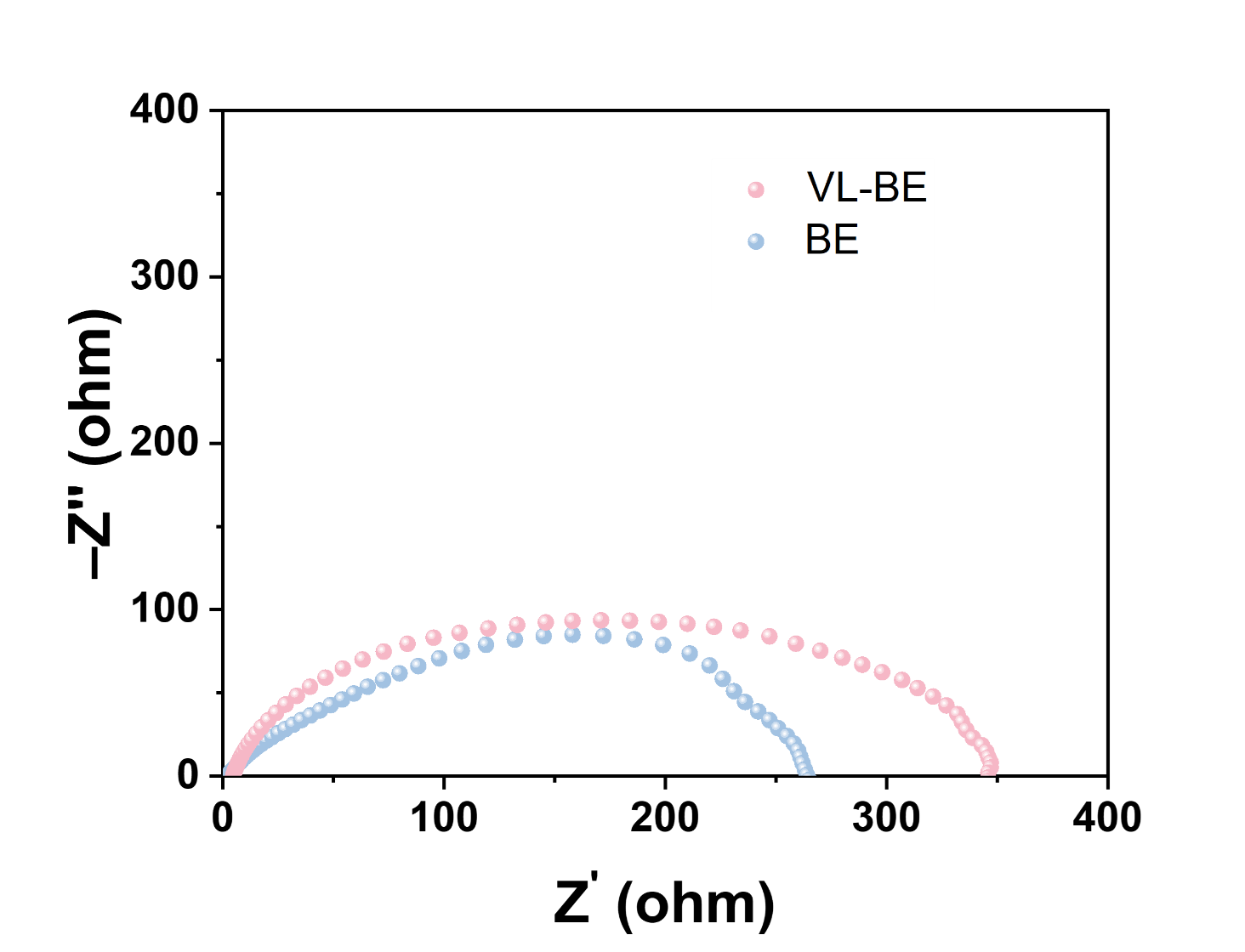
**

**Figure S5.** Nyquist plots for symmetrical batteries in different electrolytes..


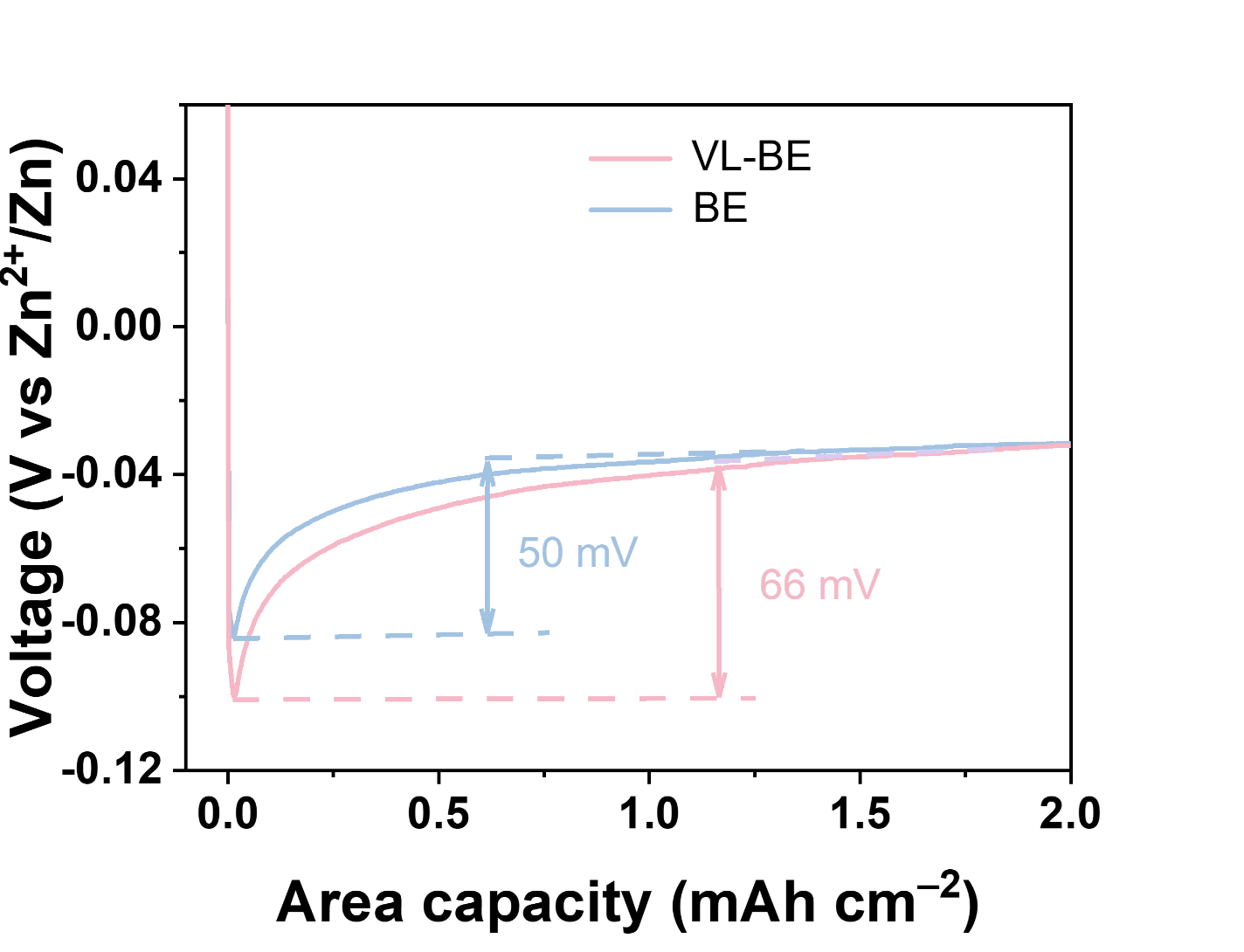


**Figure S6.** Voltage profiles during initial Zn deposition at 1 mA cm^−2^ on Zn foil in BE and VL-BE electrolytes.


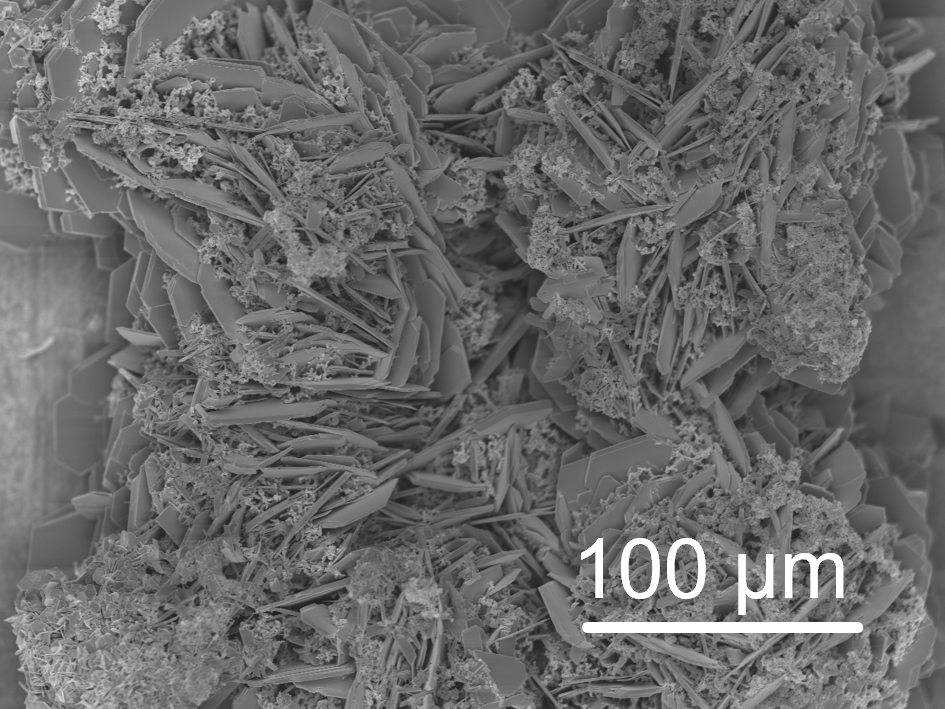


**Figure S7.** SEM image of Zn foil immersed in BE.


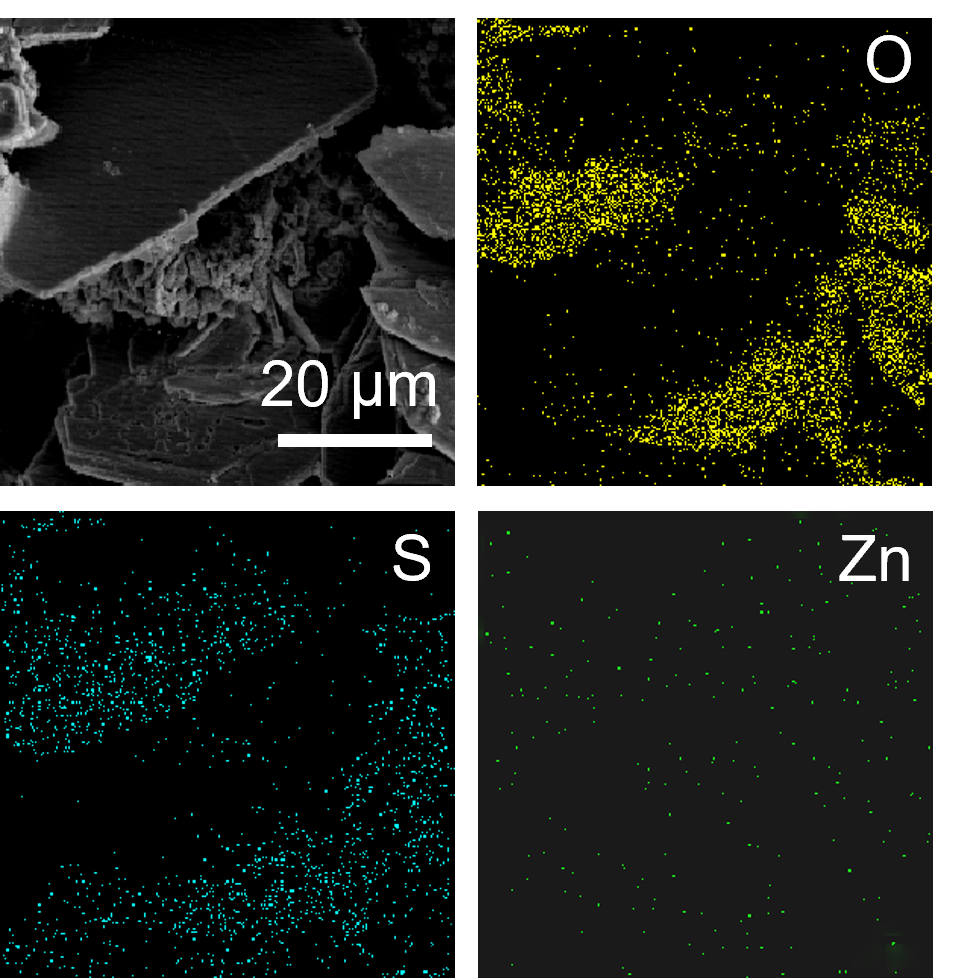


**Figure S8.** Elemental distribution analysis of Zn foil immersed in BE.

**
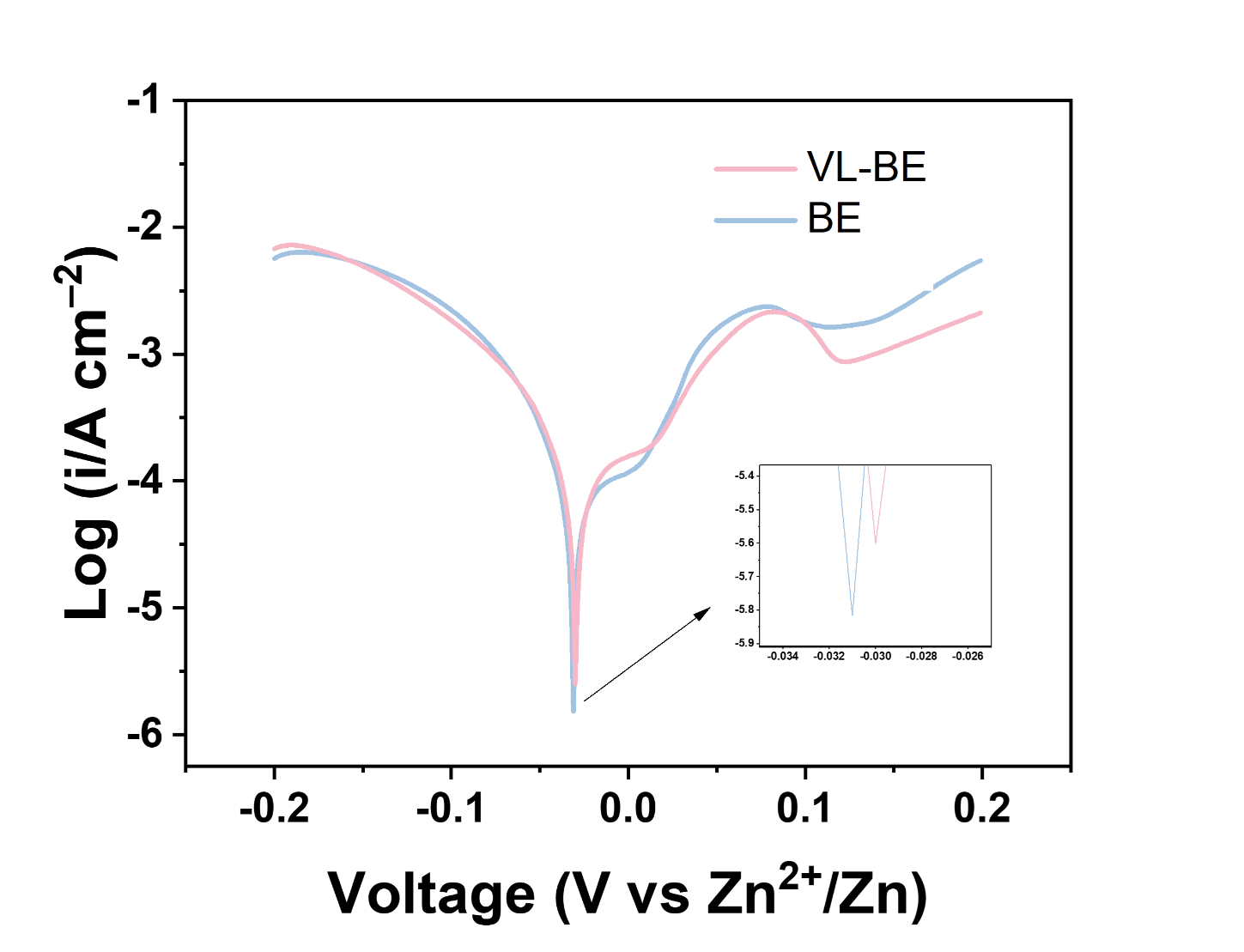
**

**Figure S9.** Tafel curves for BE and VL-BE.


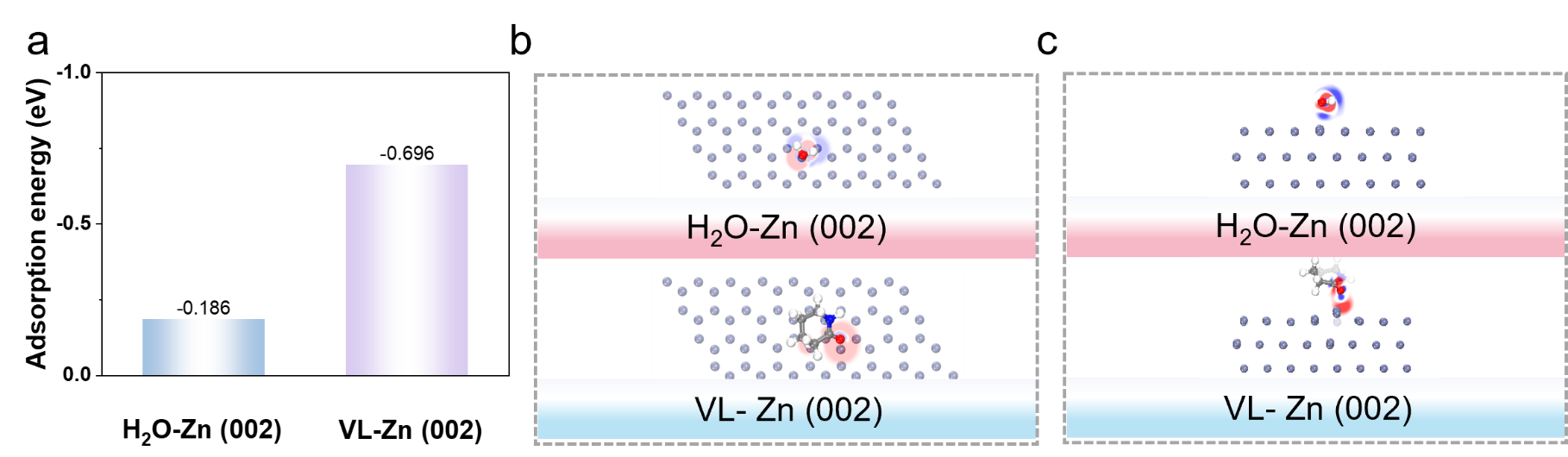


**Figure S10.** (a) Computed adsorption energies for H_2_O and VL with Zn electrode surface. Optimized structure for H_2_O and VL adsorbed on the surfaces of Zn from (b) top and (c) side view.


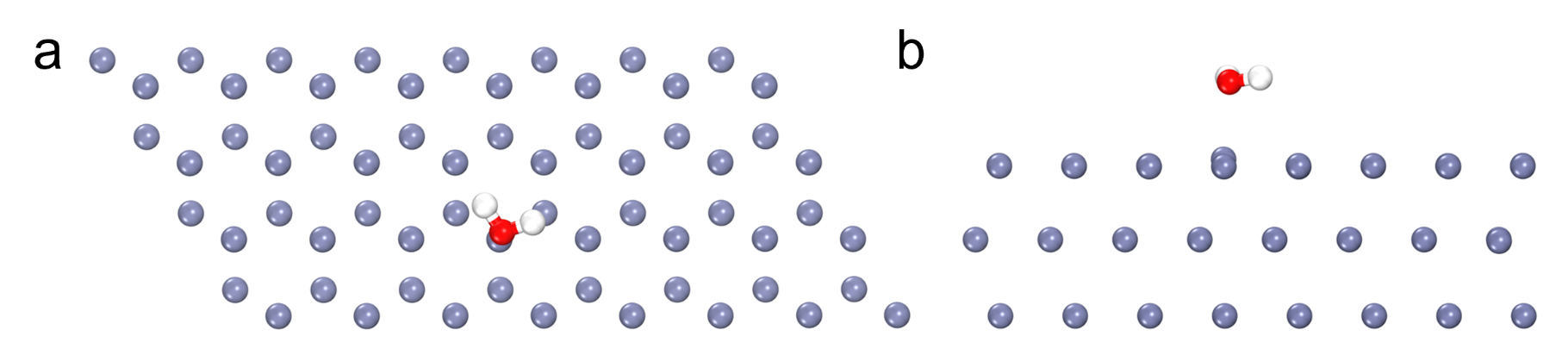


**Figure S11.** Optimized configuration of one H_2_O absorbed on Zn surface.


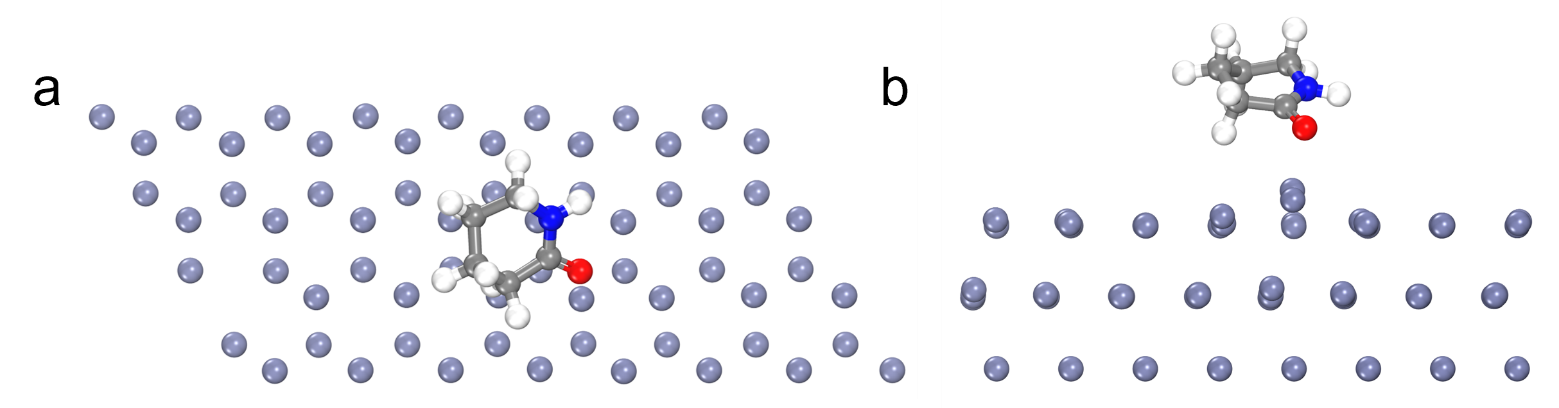


**Figure S12.** Optimized configuration of one VL absorbed on Zn surface.


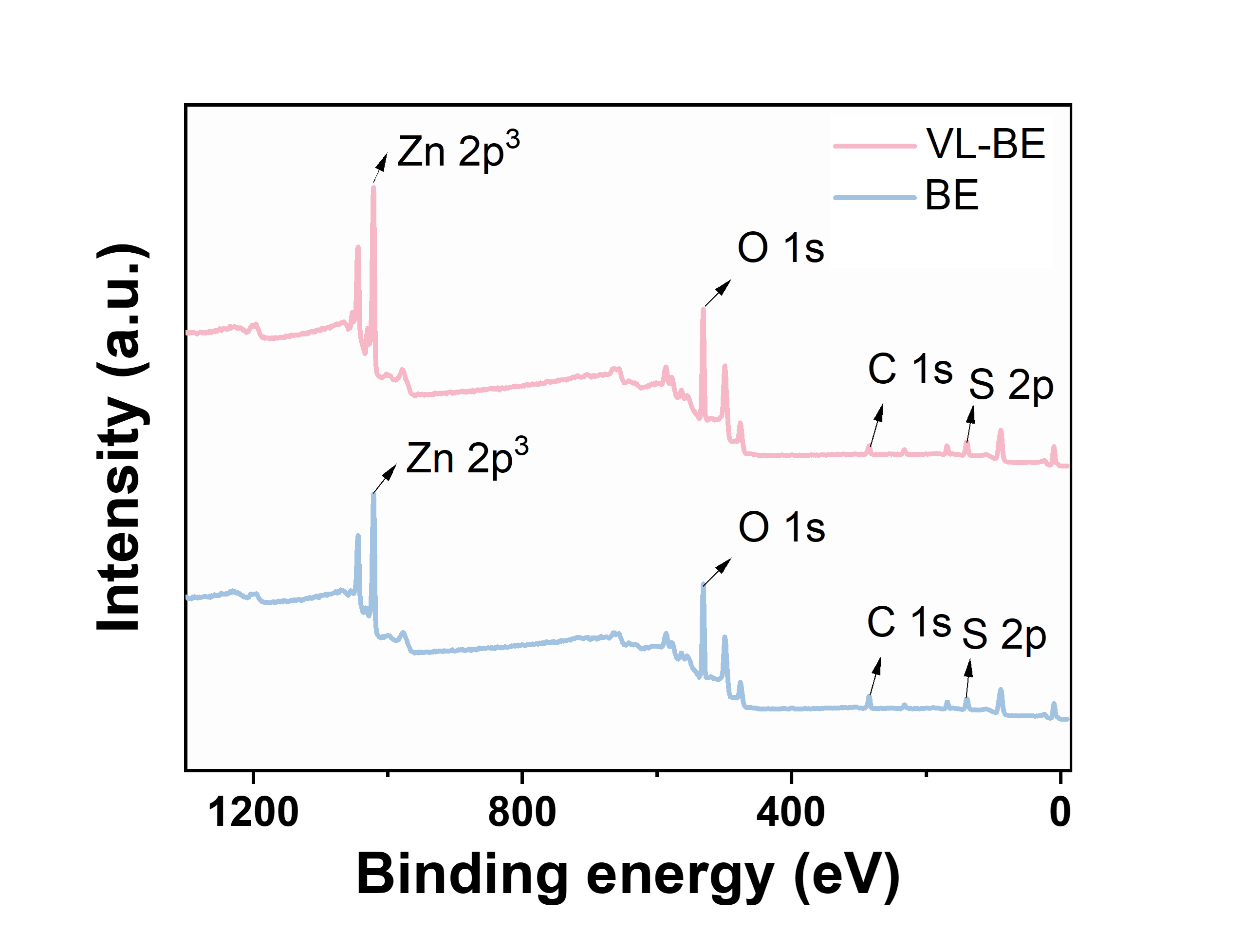


**Figure S13.** XPS spectra of Zn surface immersed in BE and VL-BE.


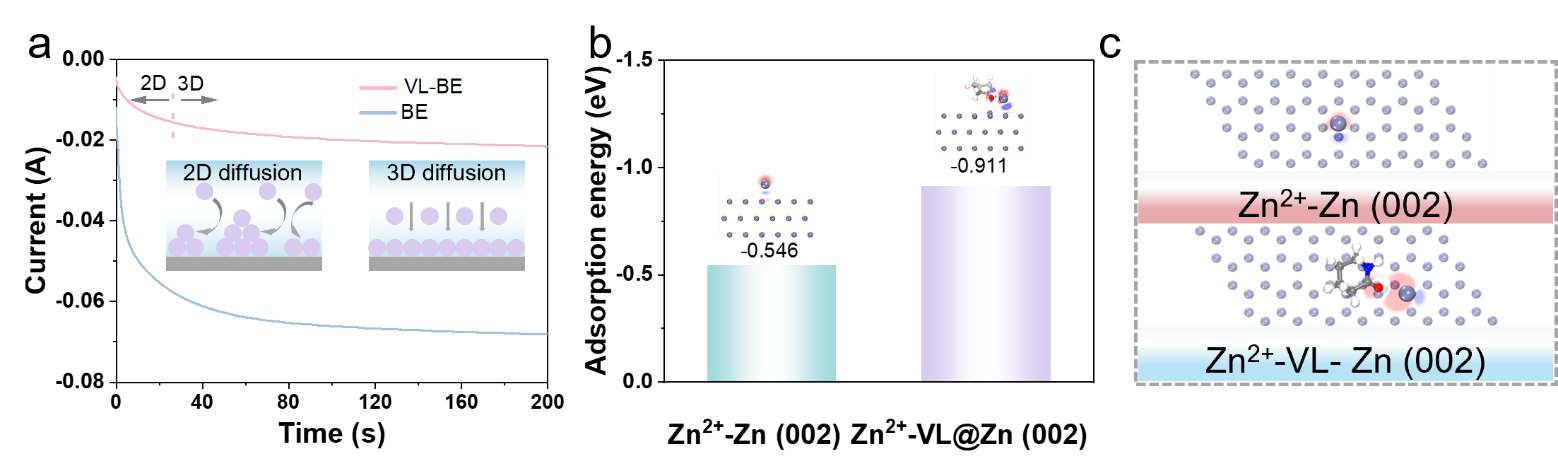


**Figure S14.** (a) CA curves for BE and VL-BE. (b) Computed adsorption energies for Zn^2+^ and Zn^2+^-VL with Zn electrode surface. (c) Optimized structure for Zn^2+^ and Zn^2+^-VL adsorbed on the surfaces of Zn from top view.


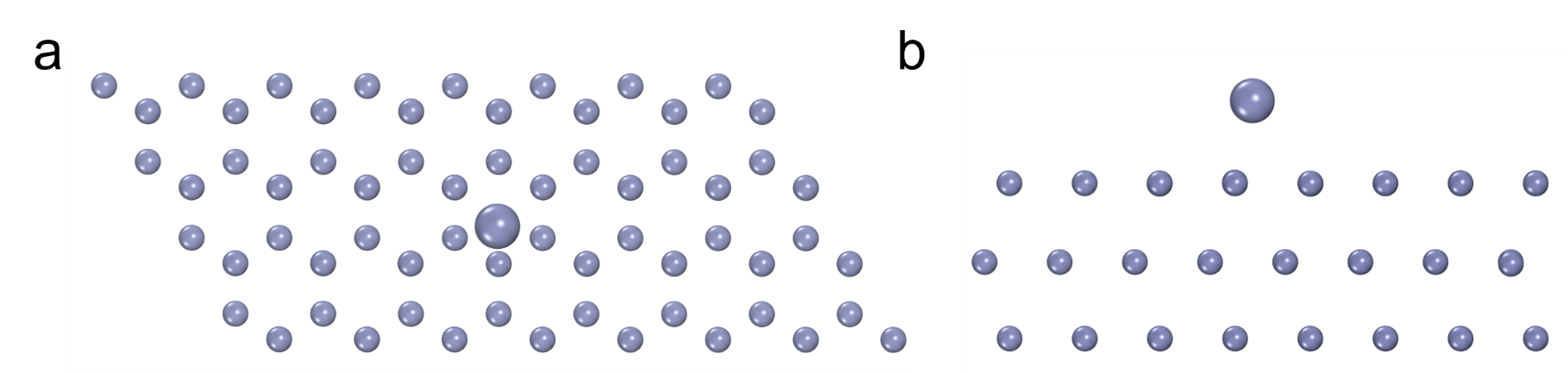


**Figure S15.** Optimized configuration of one Zn^2+^ absorbed on Zn surface


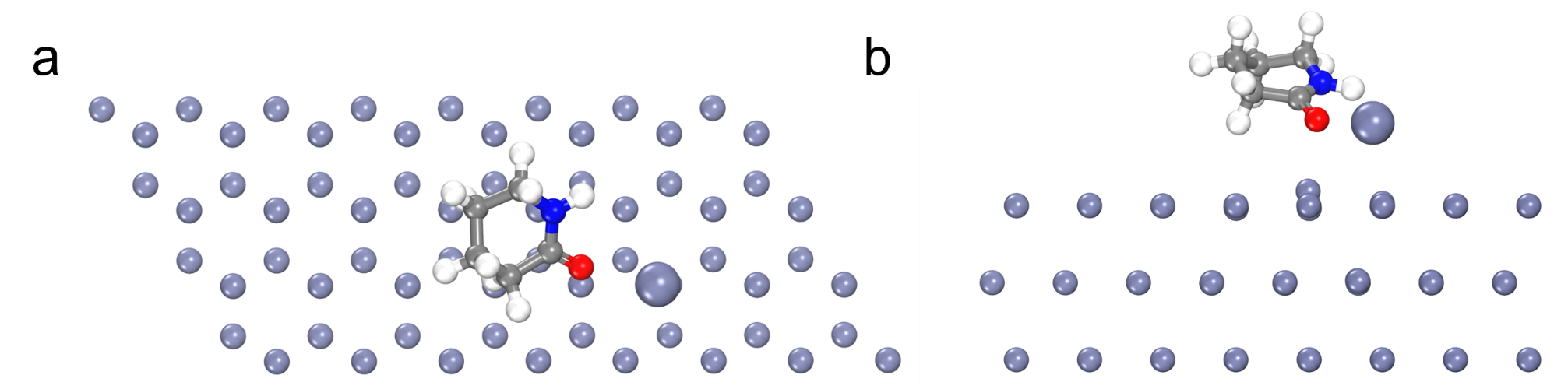


**Figure S16.** Optimized configuration of one Zn^2+^-VL absorbed on Zn surface.


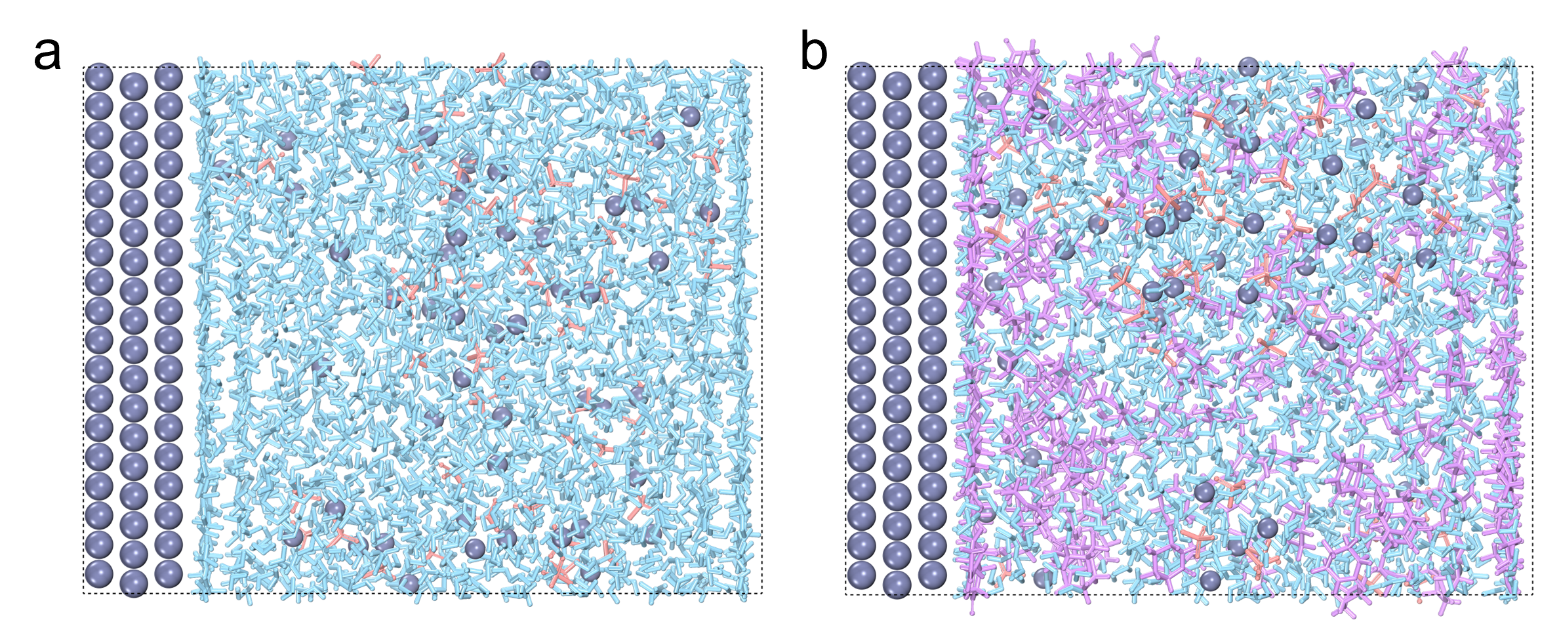


**Figure S17.** ‘Snapshots’ of the concentration distribution of H_2_O and VL on the Zn anode surface in MD simulations: (a) BE, (b) VL-BE.


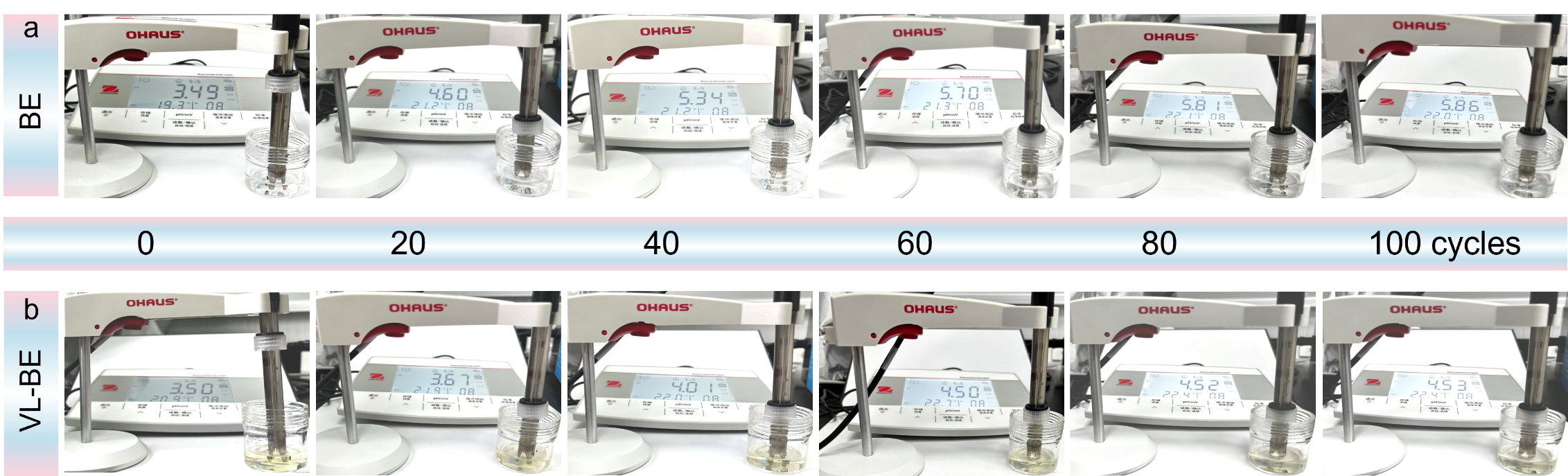


**Figure S18.** Optical images of pH test in (a) BE and (b) VL-BE.


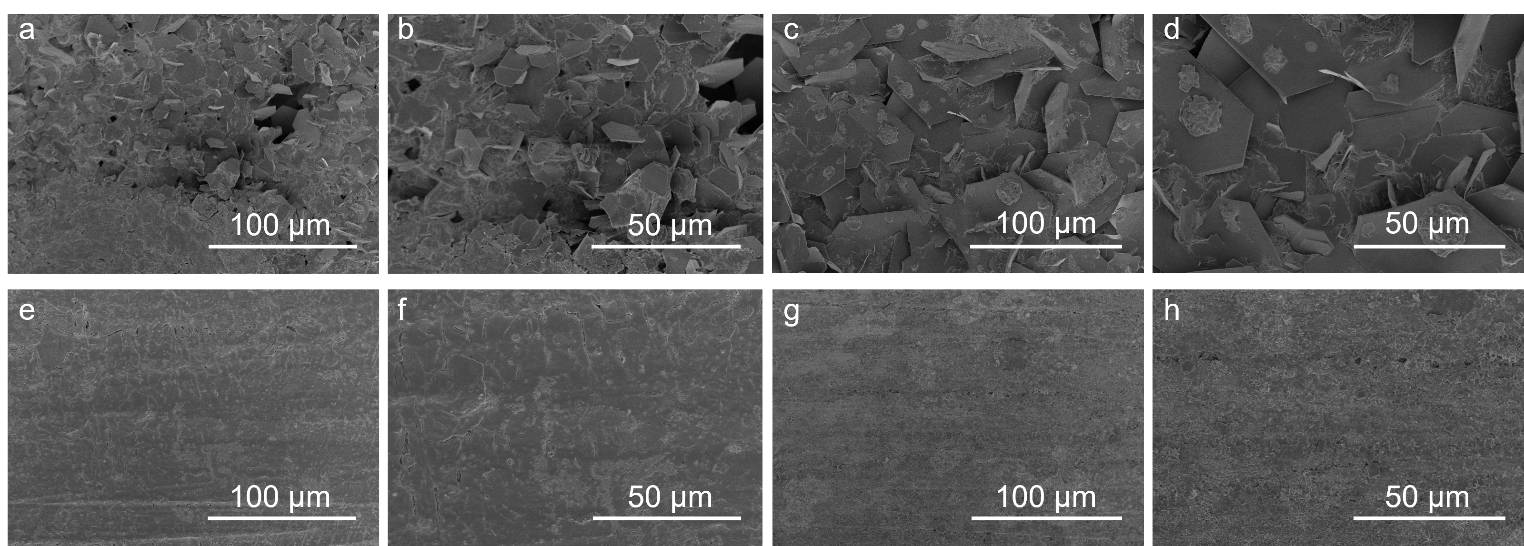


**Figure S19.** (a, b) SEM images of the Zn foil surface after depositing a capacity of 1 mAh cm^−2^ in BE. (c, d) SEM images of the Zn foil surface after depositing a capacity of 5 mAh cm^−2^ in BE. (e, f) SEM images of the Zn foil surface after depositing a capacity of 1 mAh cm^−2^ in VL-BE. (g, h) SEM images of the Zn foil surface after depositing a capacity of 5 mAh cm^−2^ in VL-BE.


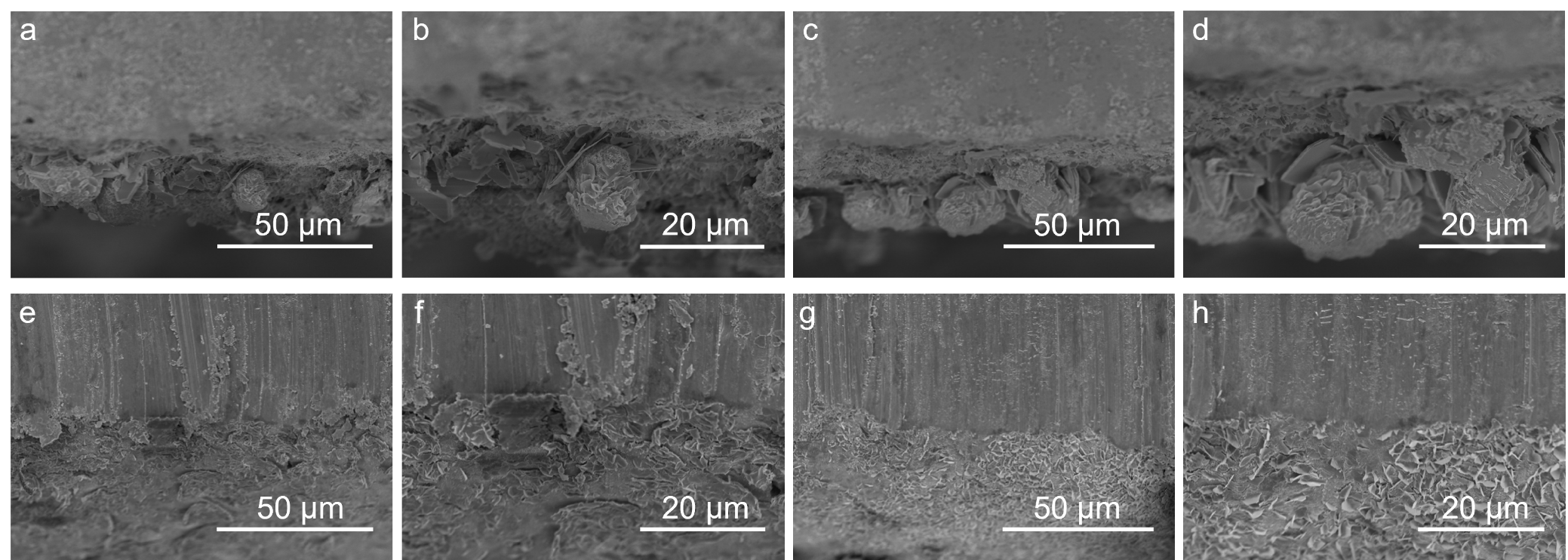


**Figure S20.** (a, b) SEM cross-sectional images of the Zn foil after depositing a capacity of 1 mAh cm^−2^ in BE. (c, d) SEM cross-sectional images of the Zn foil after depositing a capacity of 5 mAh cm^−2^ in BE. (e, f) SEM cross-sectional images of the Zn foil after depositing a capacity of 1 mAh cm^−2^ in VL-BE. (g, h) SEM cross-sectional images of the Zn foil after depositing a capacity of 5 mAh cm^−2^ in VL-BE.


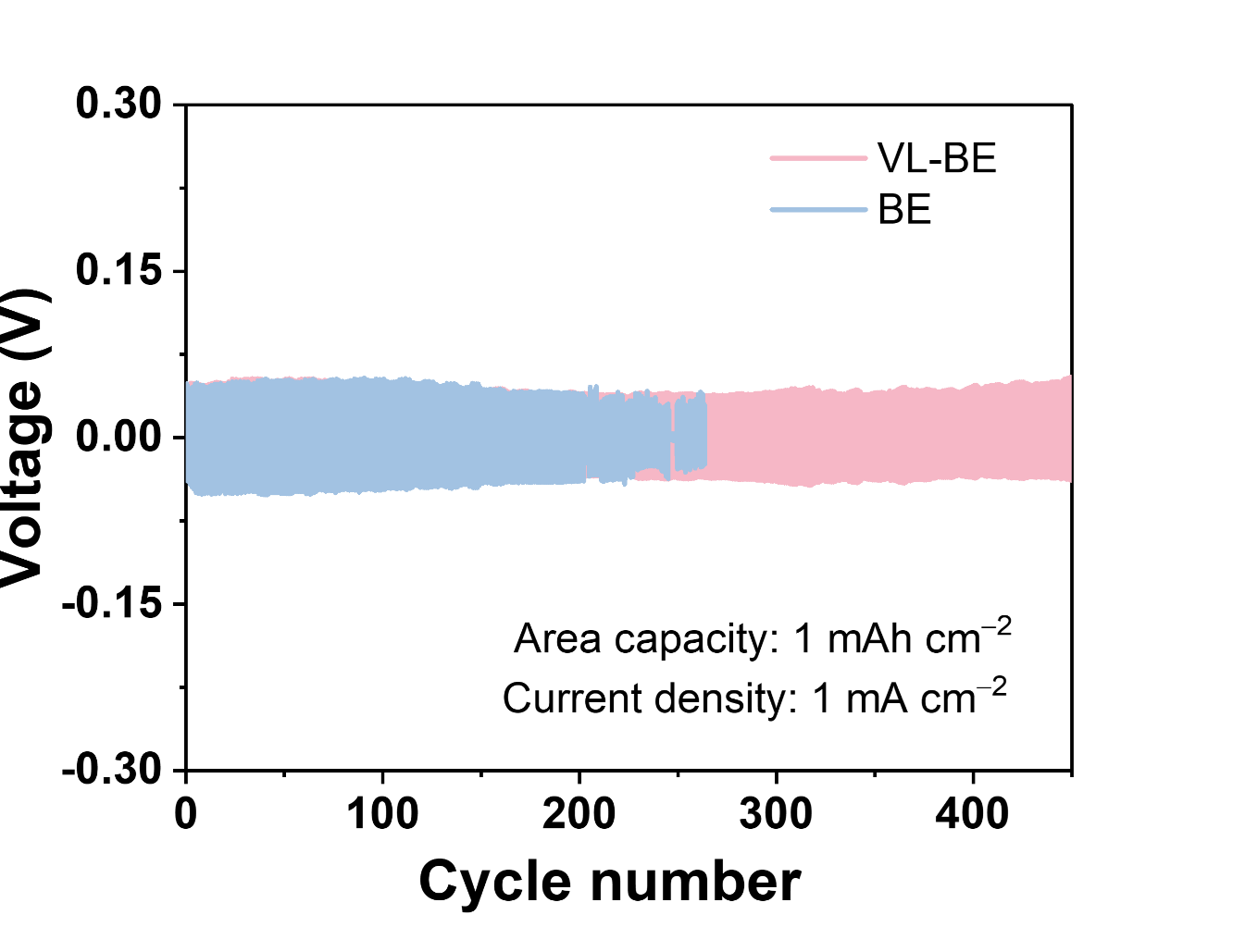


**Figure S21.** Voltage profiles for Zn symmetric cells tested in different electrolytes with current density of 1 mA cm^–2^ and capacity of 1 mAh cm^–2^.


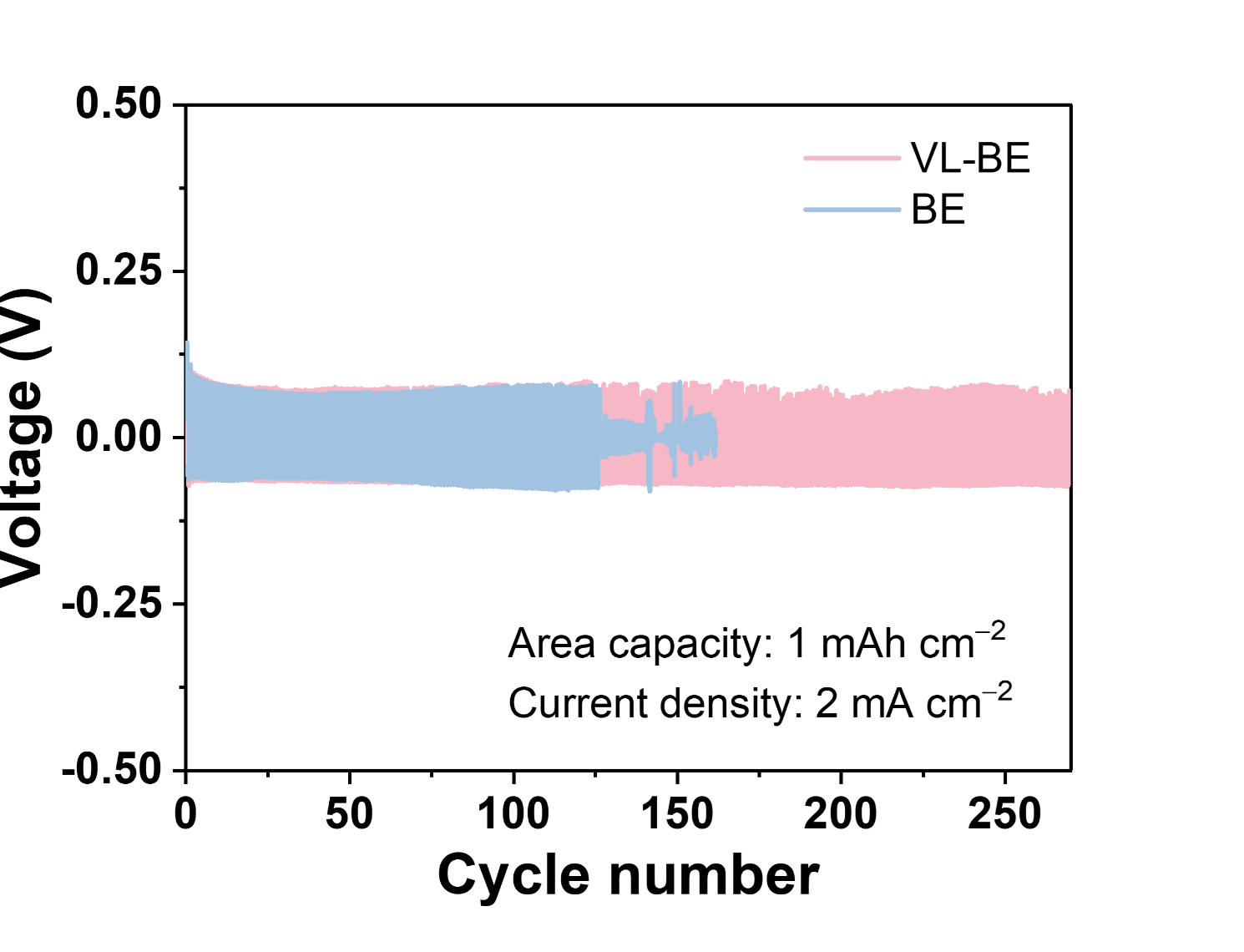


**Figure S22.** Voltage profiles for Zn symmetric cells tested in different electrolytes with current density of 2 mA cm^–2^ and capacity of 1 mAh cm^–2^.


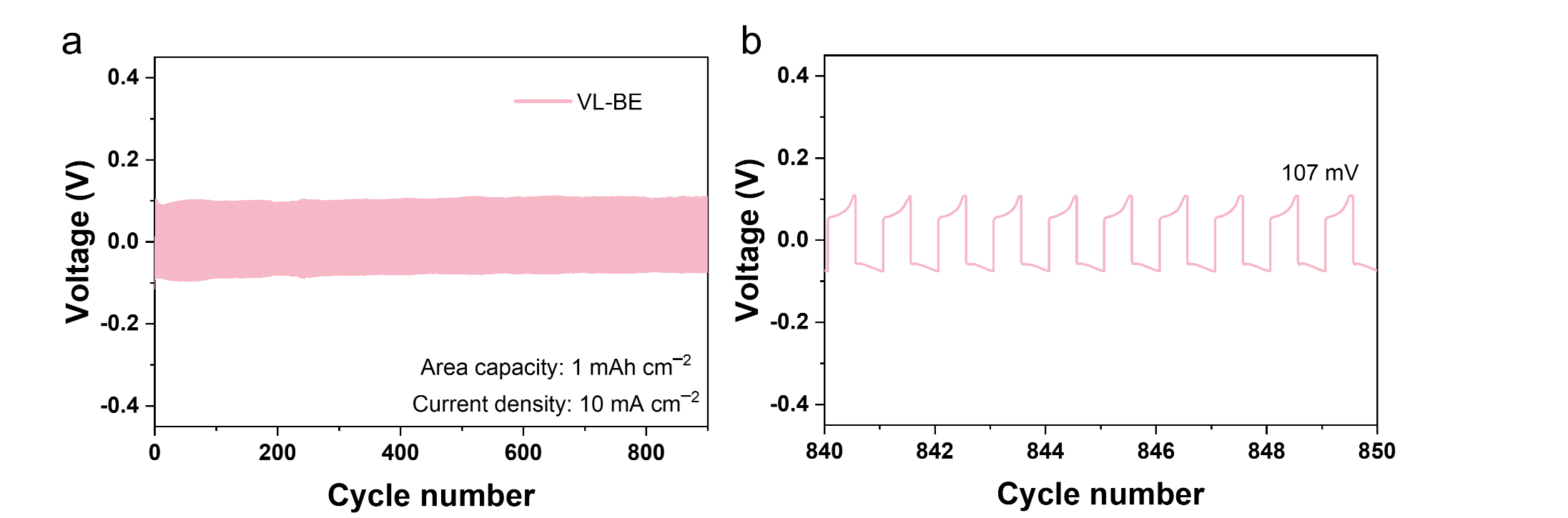


**Figure S23.** Voltage profiles for Zn symmetric batteries tested in VL-BE electrolyte with current density of 10 mA cm^–2^ and capacity of 1 mAh cm^–2^.


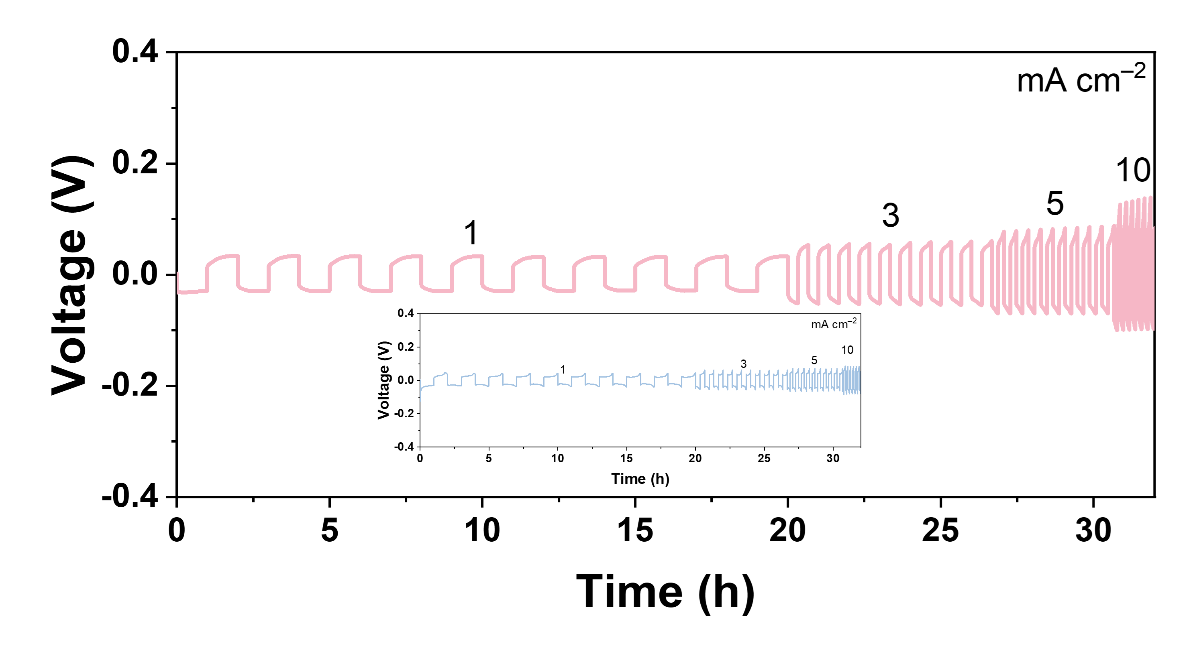


**Figure S24.** Rate performance of BE and VL-BE with a capacity of 1 mAh cm^–2^.


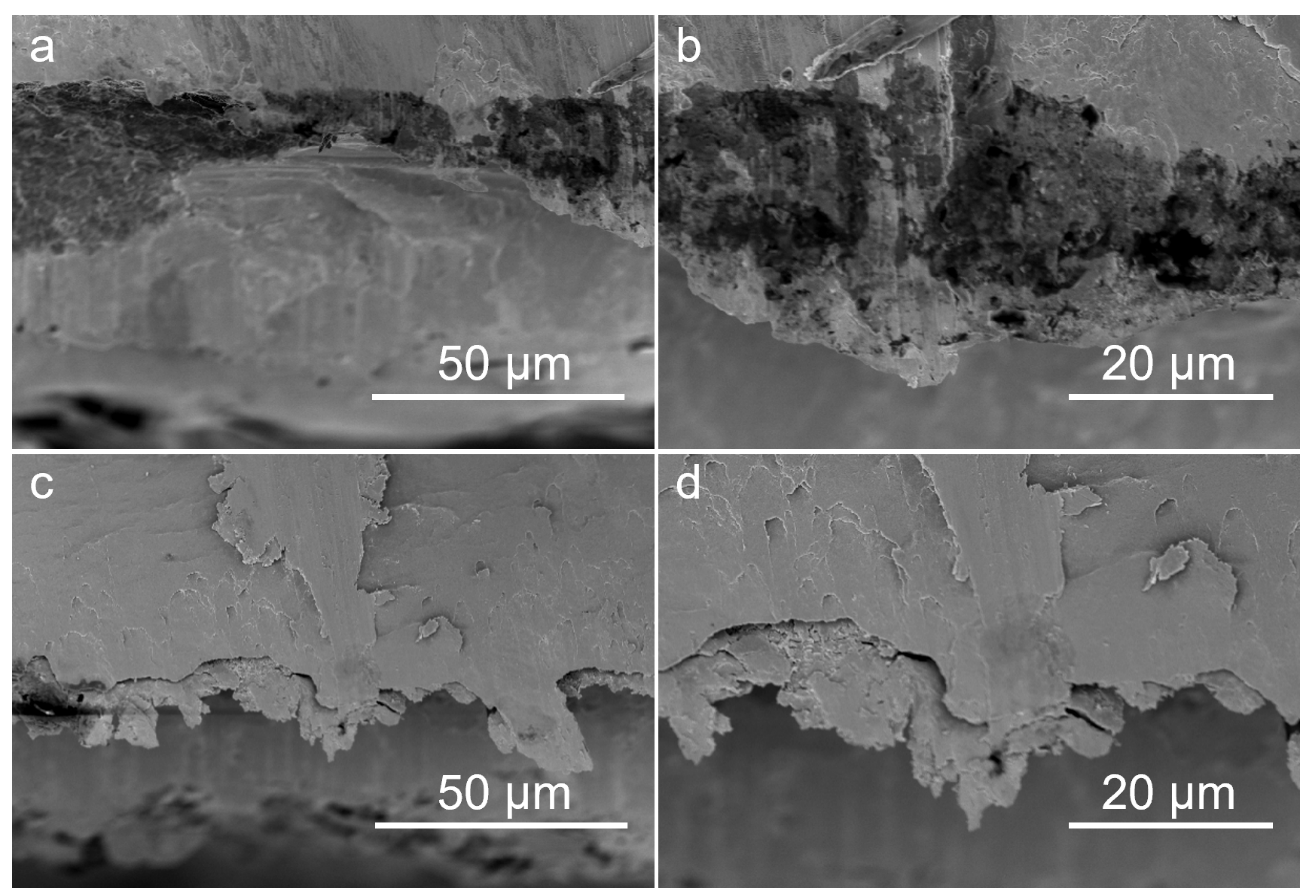


**Figure S25.** SEM cross-sectional images of the Zn foil after 100 cycles in (a, b) BE and (c, d) VL-BE.


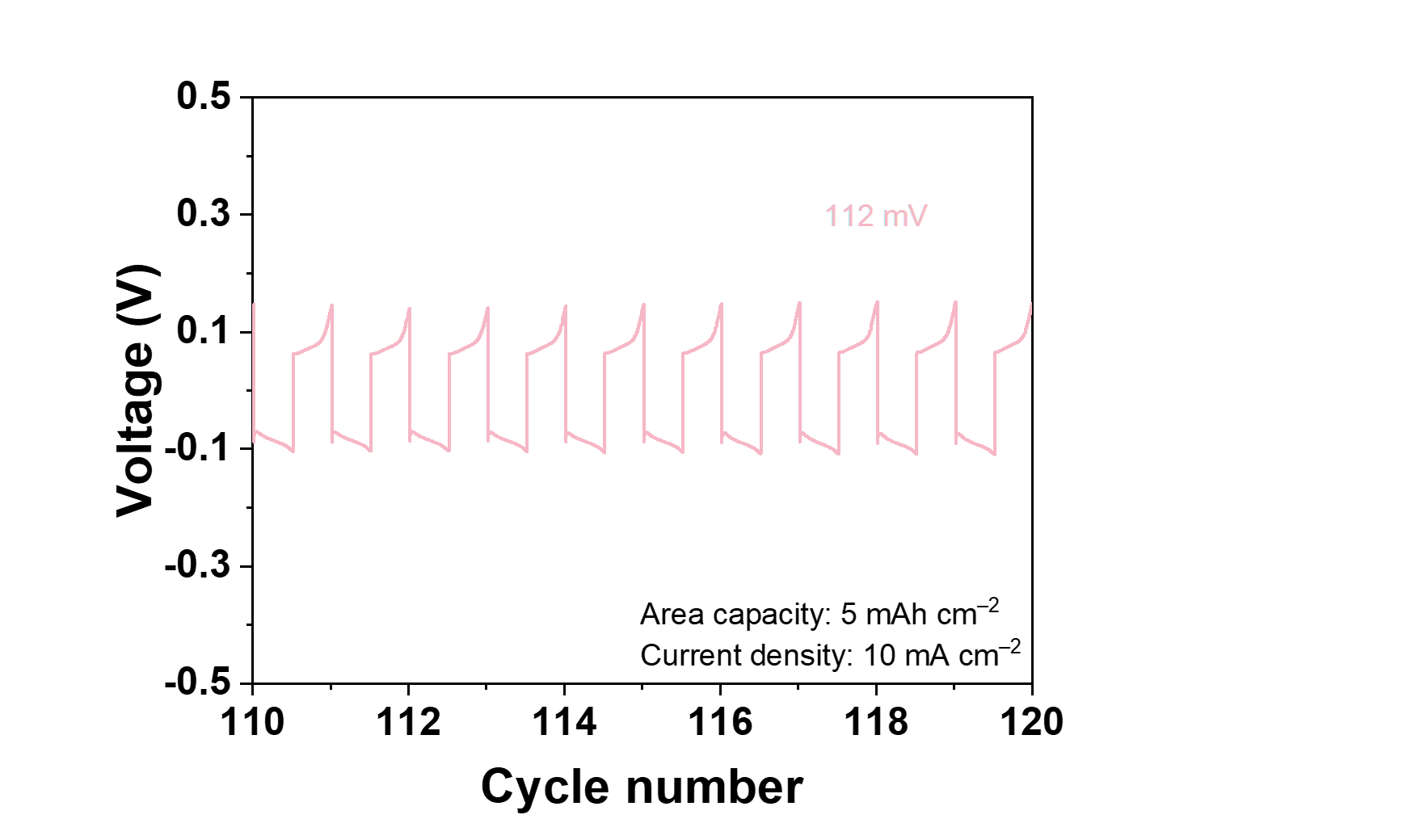


**Figure S26.** Voltage profiles for Zn symmetric battery tested in VL-BE electrolyte with current density of 10 mA cm^–2^ and capacity of 5 mAh cm^–2^.


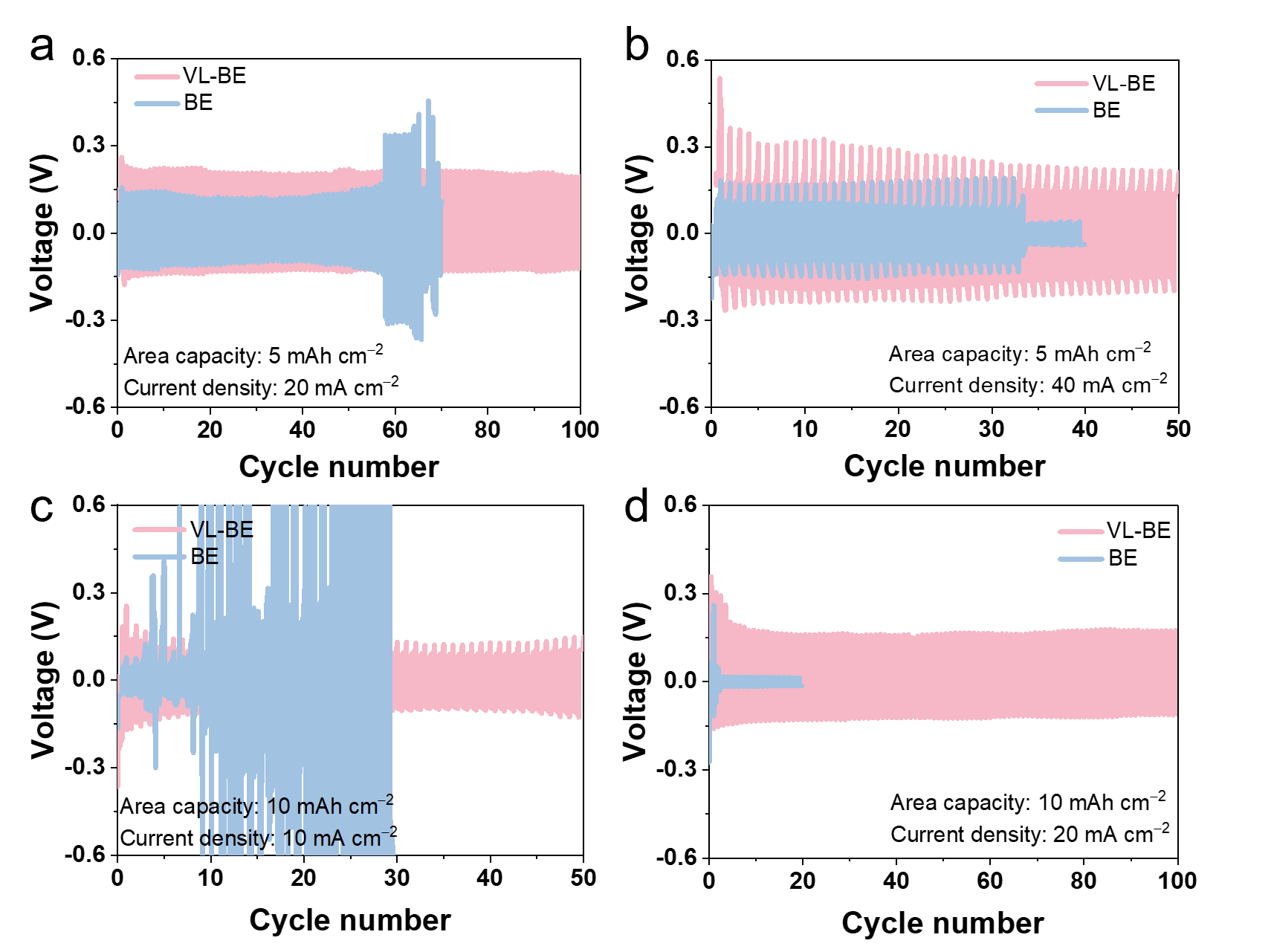


**Figure S27.** Voltage profiles for Zn symmetric cells tested in different electrolytes with various current densities and area capacities. (a) 20 mA cm^–2^ and 5 mAh cm^–2^. (b) 40 mA cm^–2^ and 5 mAh cm^–2^. (c) 10 mA cm^–2^ and 10 mAh cm^–2^. (d) 20 mA cm^–2^ and 10 mAh cm^–2^.


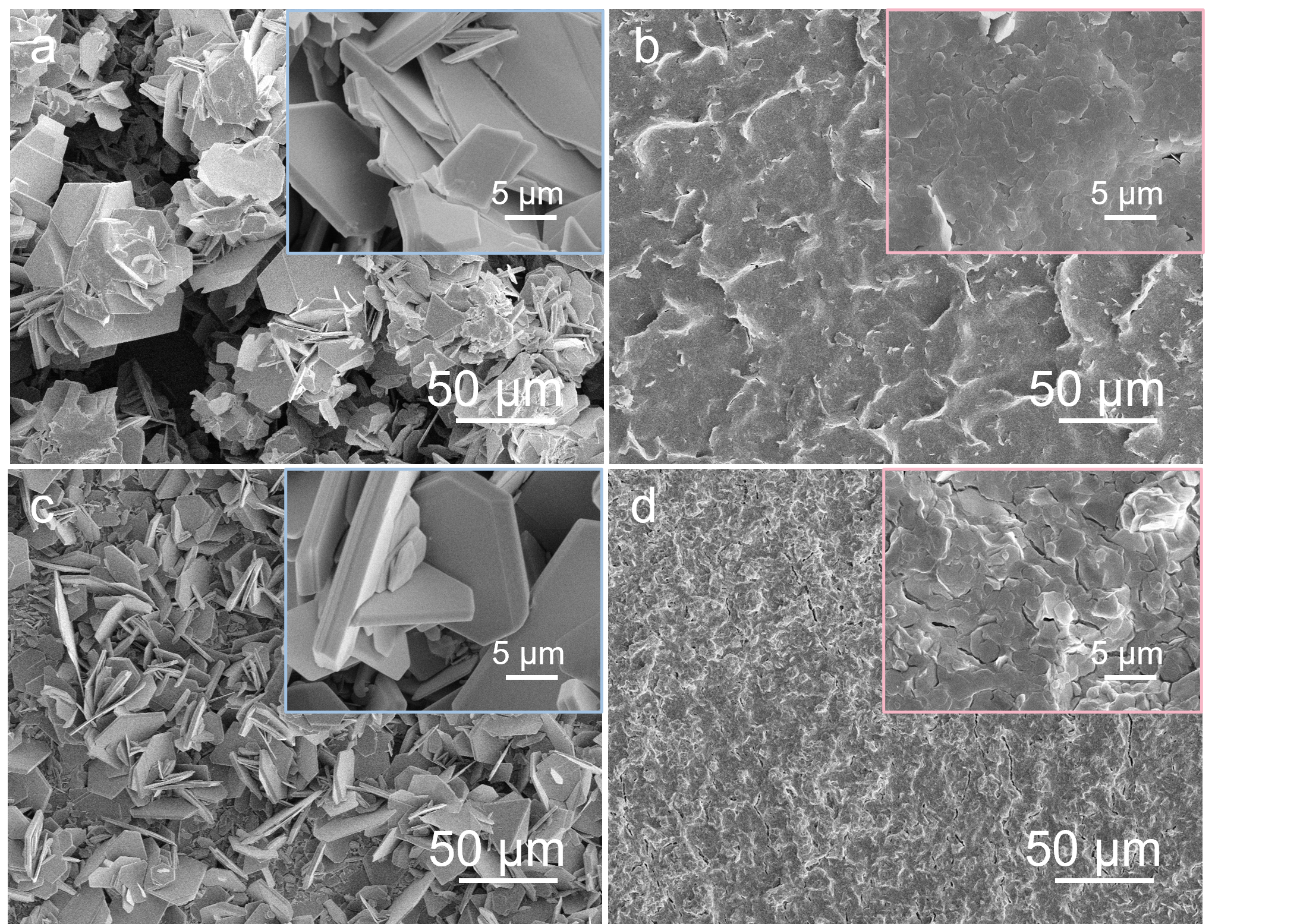


**Figures28.** SEM images of Zn anodes in different electrolytes after 50 cycles. (a) BE at 20 mA cm^–2^ and 5 mAh cm^–2^. (b) VL-BE at 20 mA cm^–2^ and 5 mAh cm^–2^. (c) BE at 40 mA cm^–2^ and 5 mAh cm^–2^. (d) VL-BE at 40 mA cm^–2^ and 5 mAh cm^–2^.


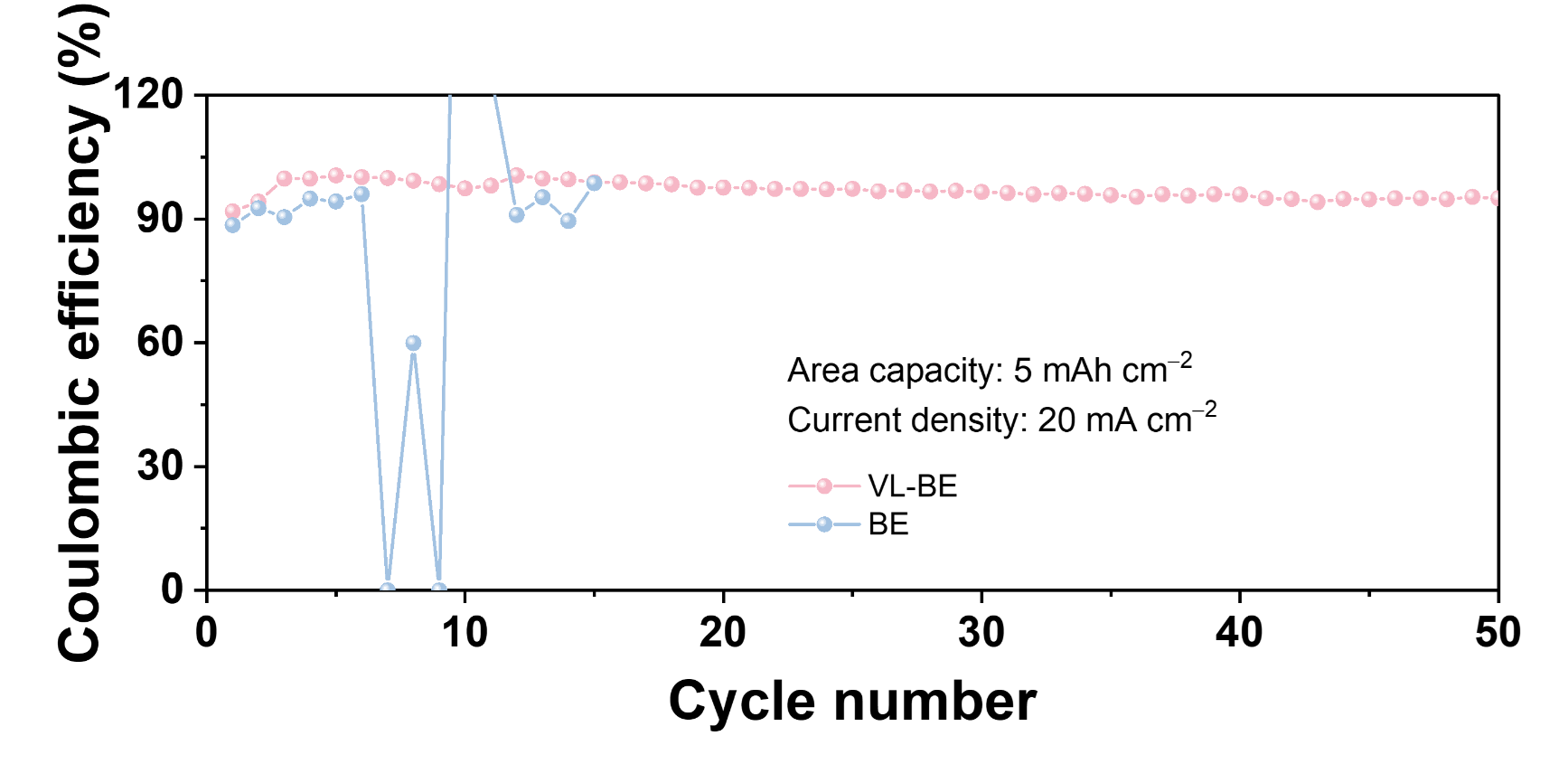


**Figure S29.** CE for Zn||Cu cells using VL-BE and BE electrolytes.


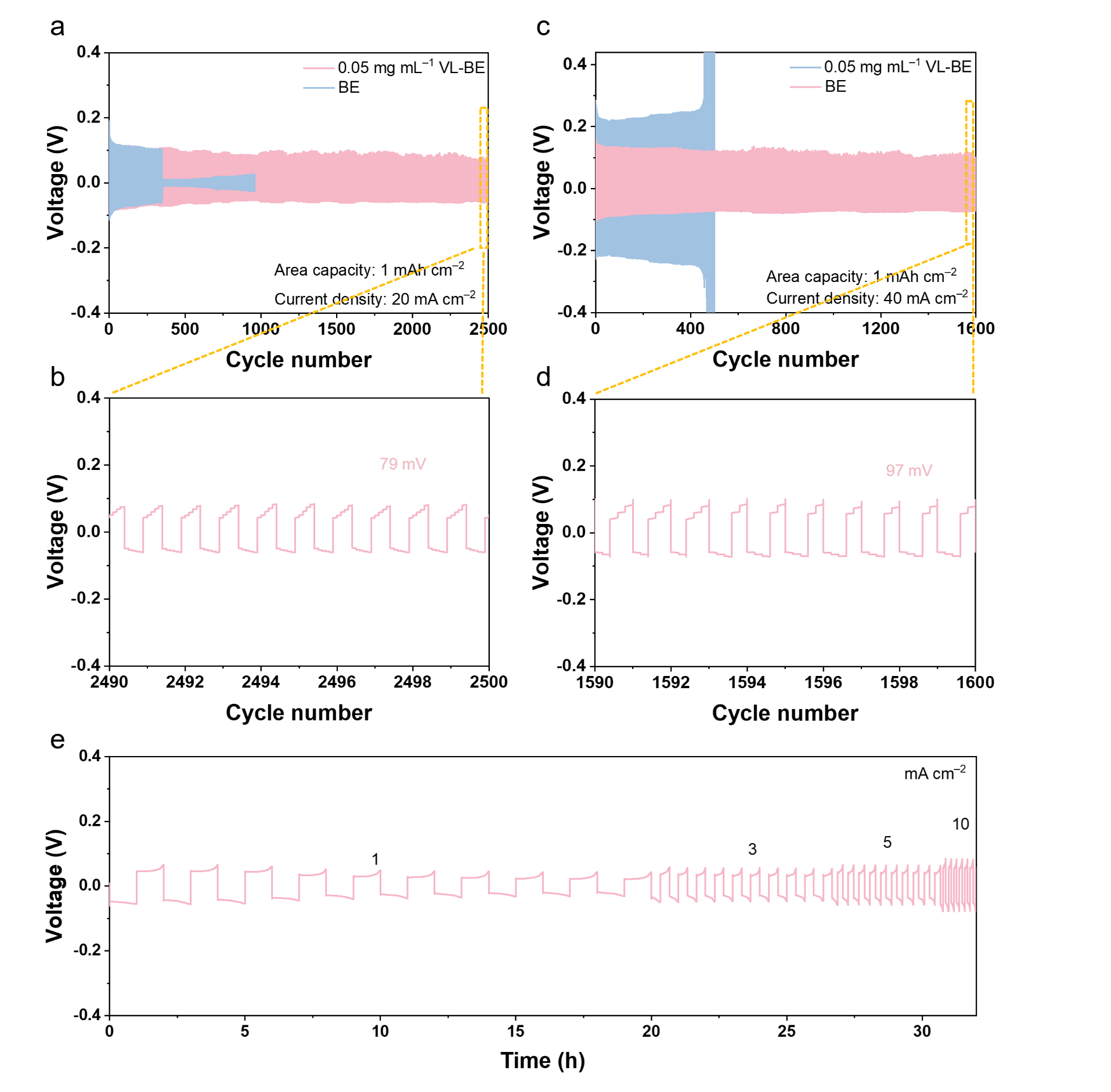


**Figure S30.** Voltage profiles for Zn symmetric batteries tested in 0.05 mg mL^–1^ VL-BE electrolyte with current density of (a, b) 20 mA cm^–2^ and (c, d) and 40 mA cm^–2^ at a capacity of 1 mAh cm^–2^. (e) Rate performance of VL-BE at a capacity of 1 mAh cm^–2^.


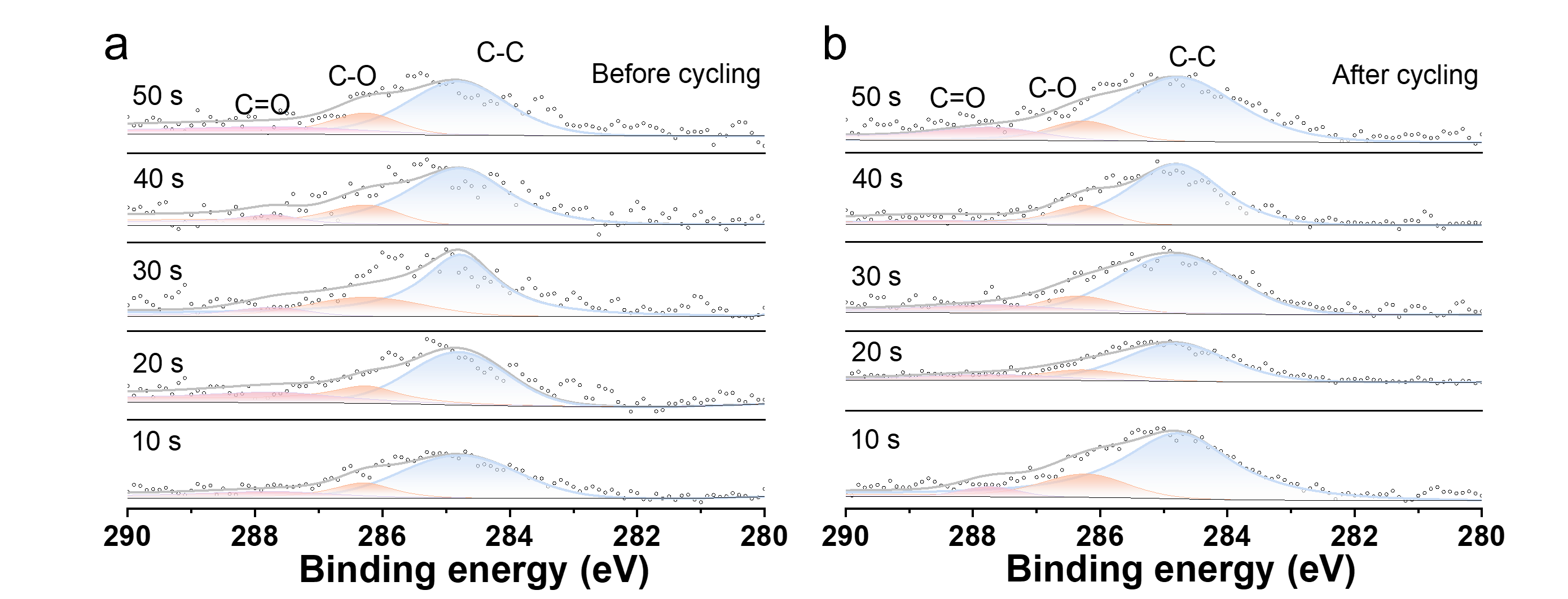


**Figure S31.** XPS spectra of Zn foil surface (a) before and (b) after cycling.


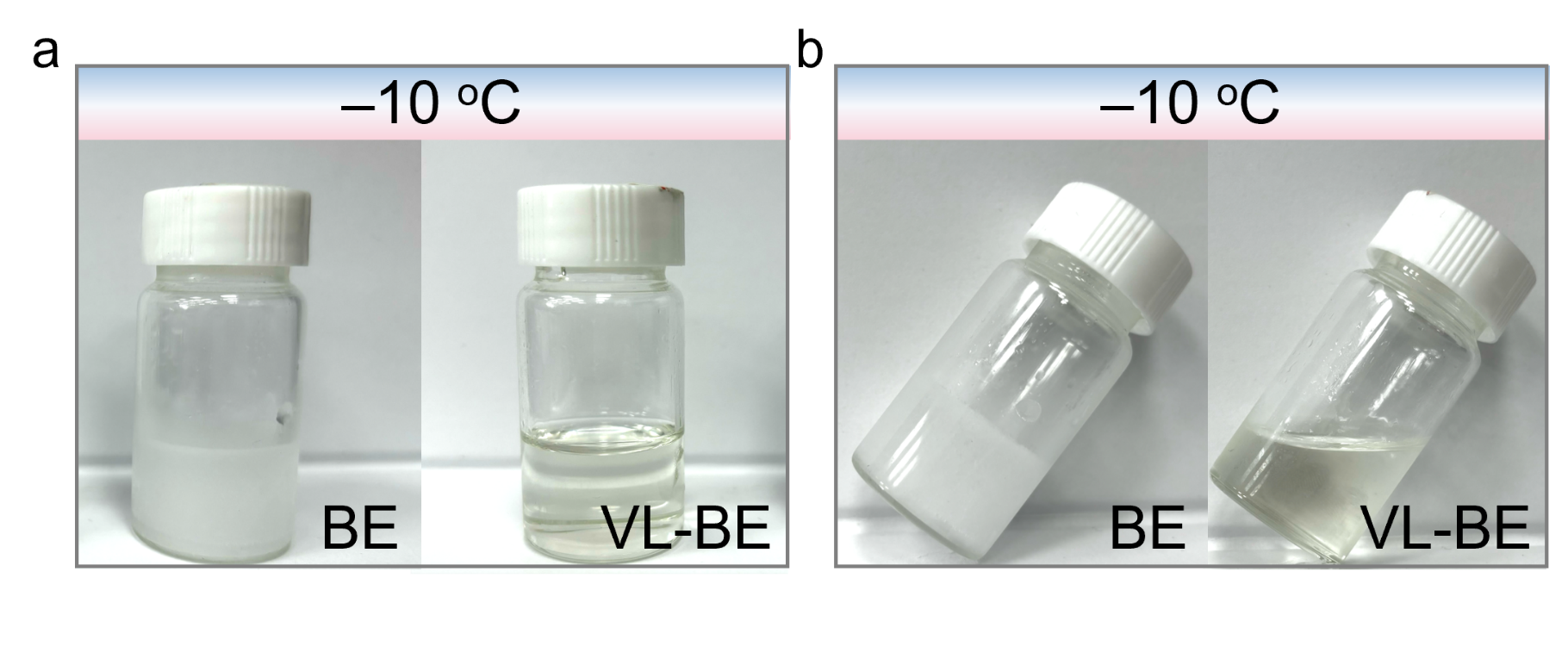


**Figure S32.** Optical images of BE and VL-BE at −10 ^o^C.


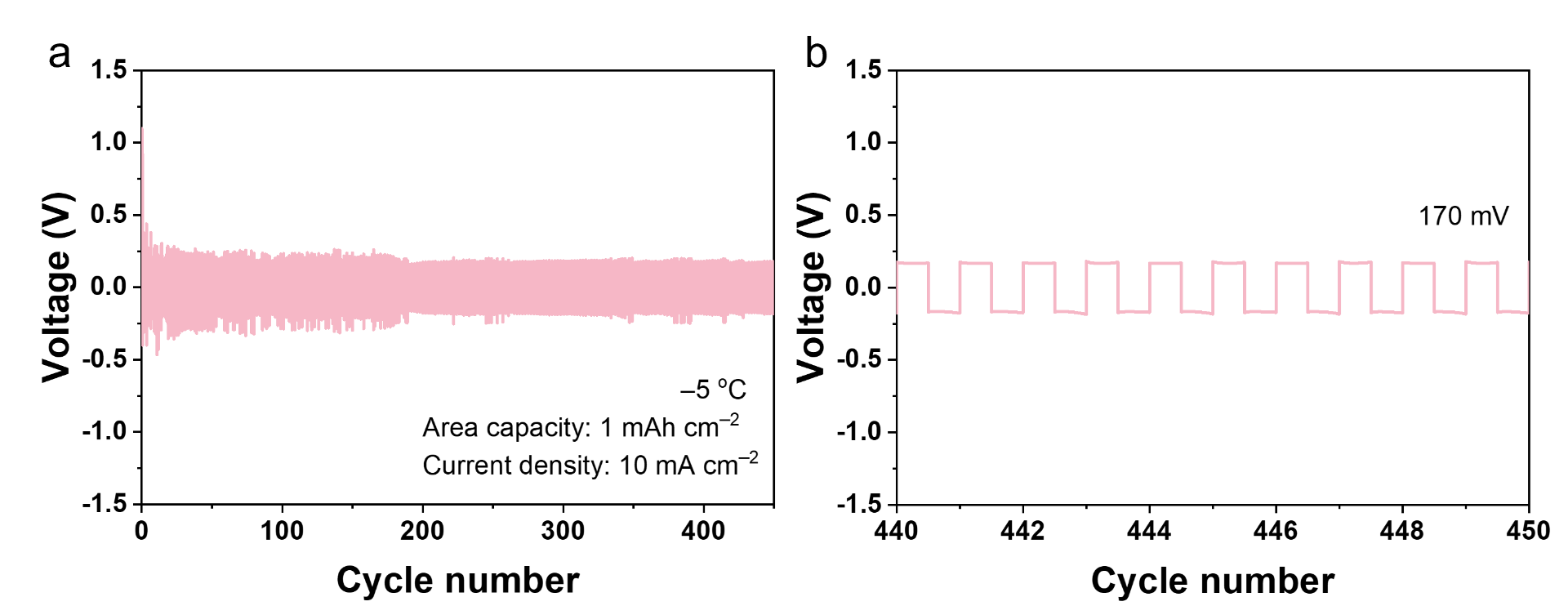


**Figure S33.** Voltage profiles for Zn symmetric battery tested in VL-BE with current density of 10 mA cm^–2^ and capacity of 1 mAh cm^–2^ at −5 ^o^C.


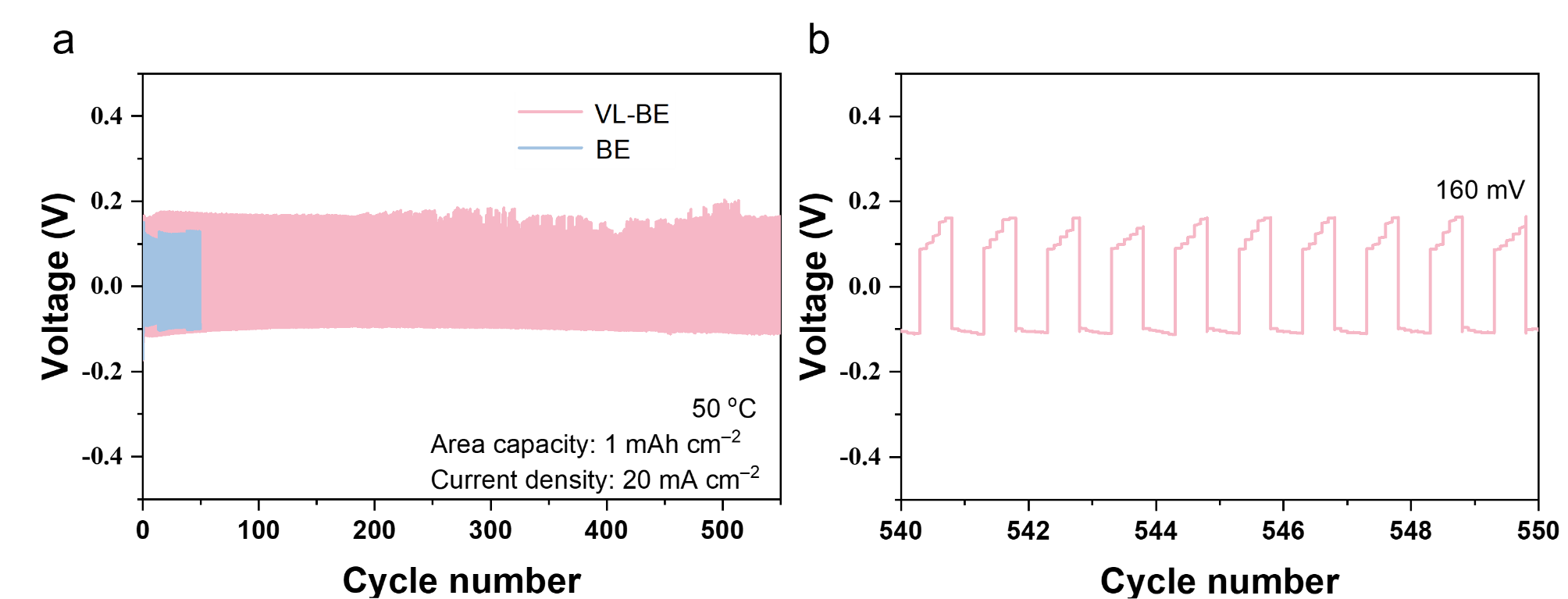


**Figure S34.** Voltage profiles for Zn symmetric batteries tested in VL-BE and BE with current density of 20 mA cm^–2^ and capacity of 1 mAh cm^–2^ at 50 ^o^C..


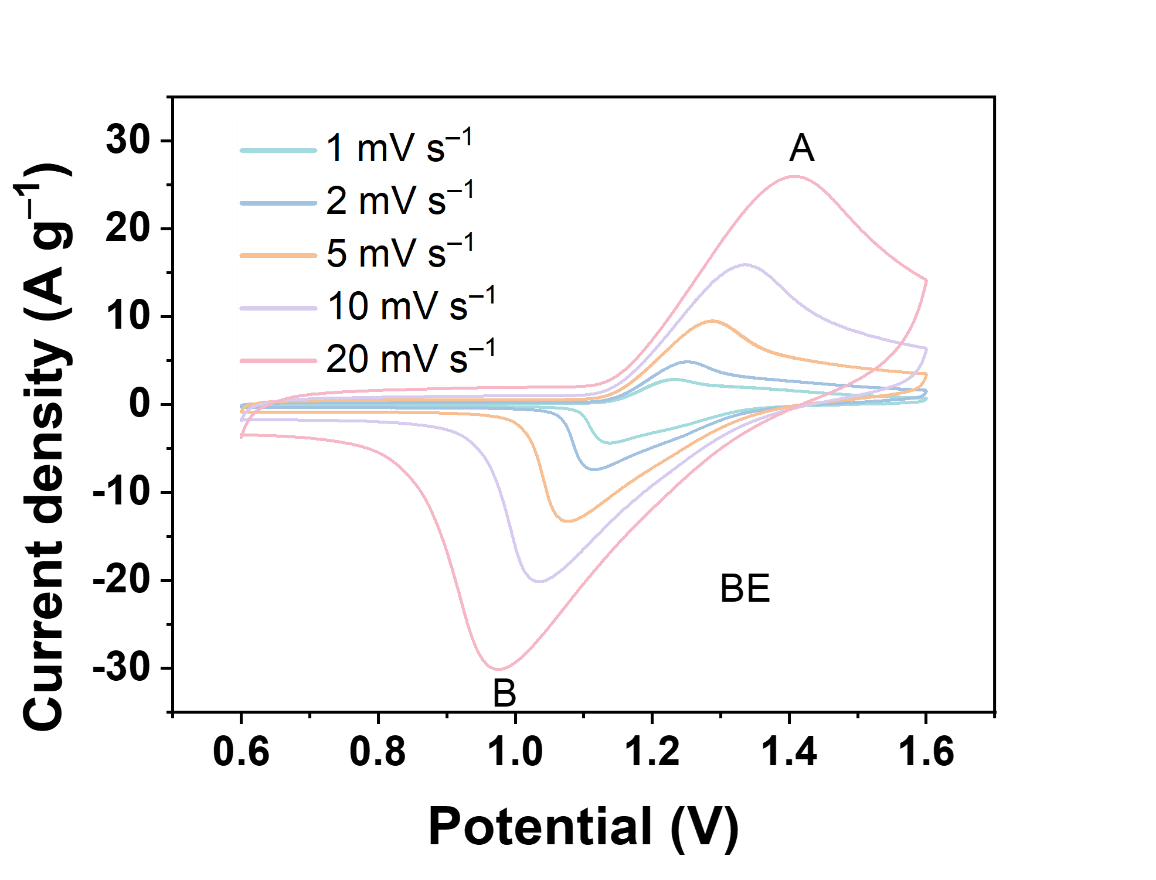


**Figure S35.** CV curves of BE determined from 0.6 to 1.6 V at various scan rates.


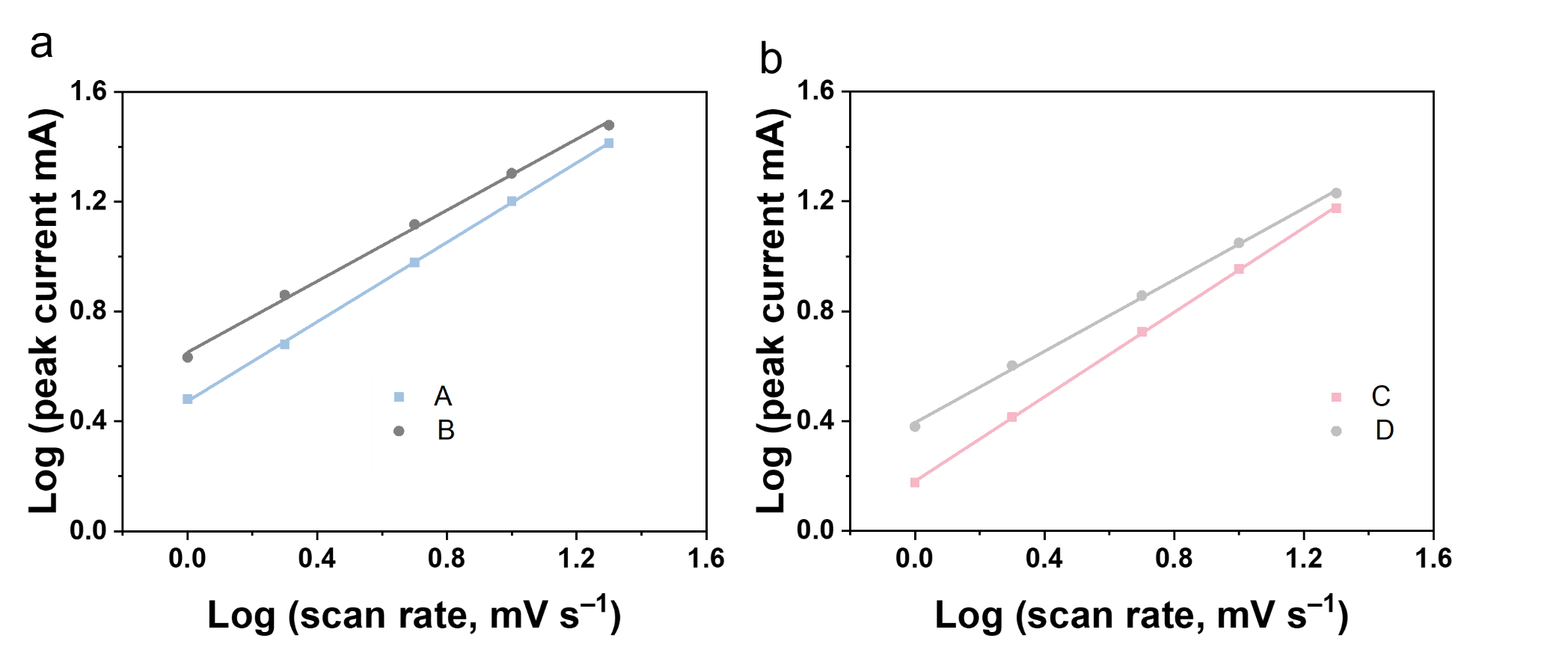


**Figure S36.** Log(i)-Log(v) curves (Figure 4h and S32-value determination).


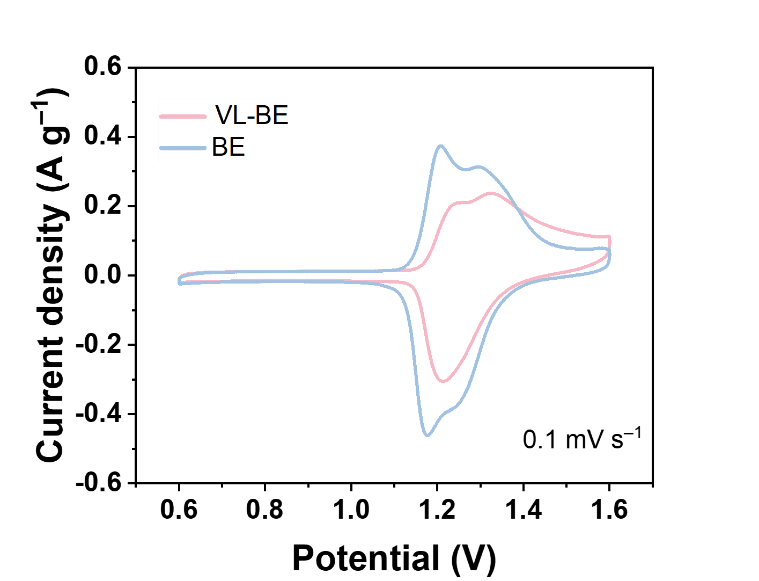


**Figure S37.** CV curves of BE and VL-BE determined from 0.6 to 1.6 V at 0.1 mV s⁻^1^.


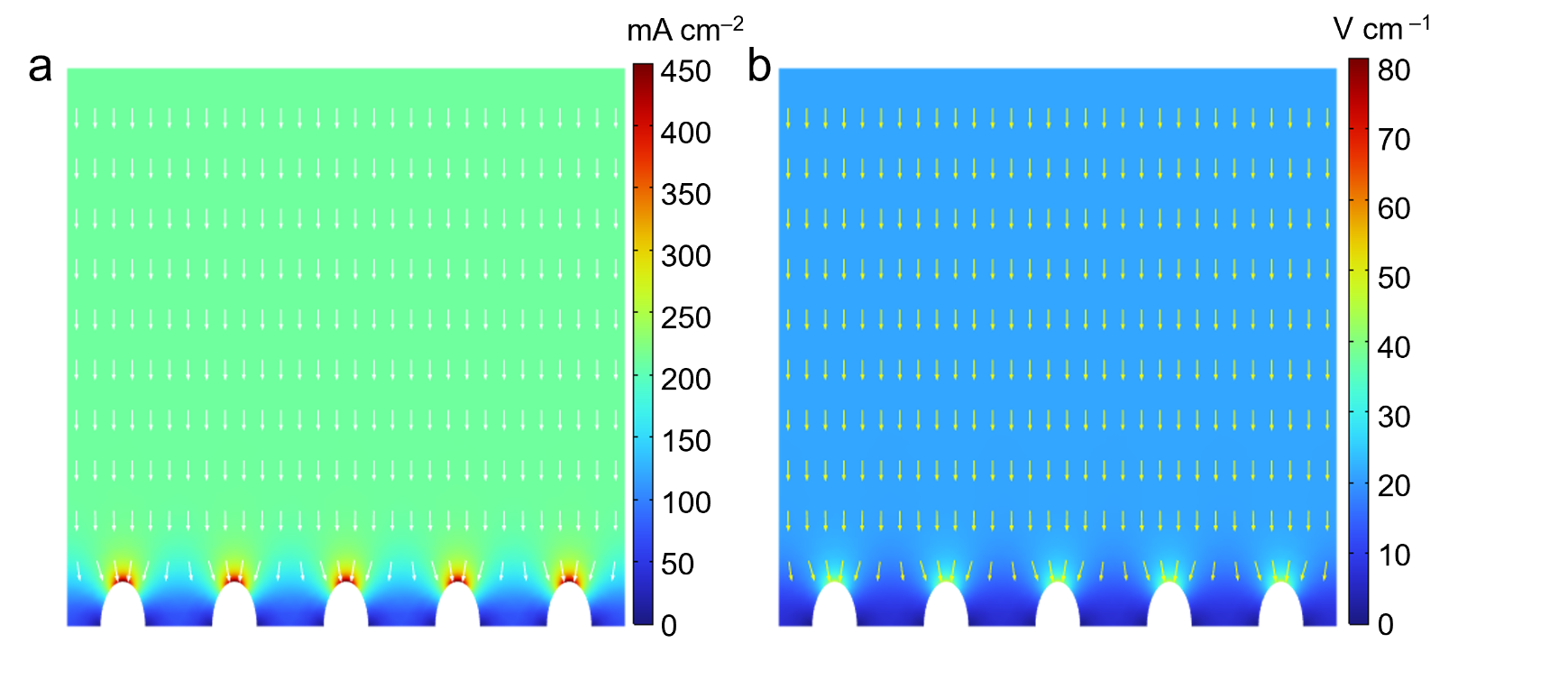


**Figure S38.** (a) Simulation of current density distribution for the Zn anodes in BE. (b) Models for the relative intensity distributions of localized electric field for the Zn anodes in BE during Zn nucleation.

# References

1. M. Frisch, G. Trucks, H. Schlegel, G. Scuseria, M. Robb, J. Cheeseman, G. Scalmani, V. Barone, G. Petersson, H. Nakatsuji, *Gaussian Inc. Wallingford CT* **2016**, *1*, 572.
2. S. J. Clark, M. D. Segall, C. J. Pickard, P. J. Hasnip, M. I. Probert, K. Refson, M. C. Payne, *Z Krist-CRryst Mater.* **2005**, *220*, 567–570.
3. J. P. Perdew, K. Burke, M. Ernzerhof, *Phys. Rev. lett.* **1996**, *77*, 3865.
4. P. Hasnip, C. Pickard, *Comput. Phys. Commun.* **2006**, *174*, 24−29.
5. J. D. Head, M. C. Zerner, *Chem. Phys. Lett.* **1985**, *122*, 264–270.
6. W. Tang, E. Sanville, G. Henkelman, *J. Phys.: Condens. Matter* **2009**, *21*, 084204.
7. A. Y. Toukmaji, J. A. Board Jr, *Comput*, *Phys. Commun.* **1996**, *95*, 73–92.
8. G. Kalibaeva, M. Ferrario, G. Ciccotti, *Mol. Phys.* **2003**, *101*, 765–778.
9. W. Chen, Y. Hu, W. Q. Lv, T. Y. Lei, X. F. Wang, Z. H. Li, M. Zhang, J. W. Huang, X. C. Du, Y. C. Yan, W. D. He, C. Liu, M. Liao, W. L. Zhang, J. Xiong, C. L. Yan, *Nat. Commun.* **2019**, *10*, 4973.
10. G. X. Li, Z. Liu, Q. Q. Huang, Y. Gao, M. Regula, D. W. Wang, L. Q. Chen, D. H. Wang, *Nat. Energy* **2018**, *3*, 1076–1083.

# Author Contributions

J. L., C. G. and P. X. conceived and designed the idea. J. L., Z. F., H. C and N. W. designed the experiments, collected and analyzed the data. Y. P., K. L., X. L., D. L., C. H. and Y. Y. papered the experiments and characterizations. C. G., P. X. and N. W. discussed the results and prepared the manuscript. N. W. wrote the manuscript. G. H. and X. P. helped with the manuscript revising and data analysis.
